# Silent witnesses: basidiomycota as forensic biomarkers in human decomposition

**DOI:** 10.3389/fmicb.2026.1774584

**Published:** 2026-07-13

**Authors:** Samantha C. Karunarathna, Saowaluck Tibpromma, Baggya Sharmali Karunarathna, Dong-Qin Dai, Jaturong Kumla, Wenhua Lu, Rekhani Hansika Perera, Tikka Dewage Chamarika Priyadarshani, Kunhiraman C. Rajeshkumar, Meimei Wang, Kalani Kanchana Hapuarachchi, Nakarin Suwannarach

**Affiliations:** 1 Center for Yunnan Plateau Biological Resources Protection and Utilization & Yunnan International Joint Laboratory of Fungal Sustainable Utilization in South and Southeast Asia, College of Biology and Food Engineering, Qujing Normal University, Qujing, China; 2 Faculty of Science, Department of Chemistry, Eastern University Sri Lanka, Chenkalady, Sri Lanka; 3 Center of Excellence in Microbial Diversity and Sustainable Utilization, Chiang Mai University, Chiang Mai, Thailand; 4 Office of Research Administration, Chiang Mai University, Chiang Mai, Thailand; 5 Zest Lanka International (Private) Limited, Polonnaruwa, Sri Lanka; 6 Faculty of Agriculture, Department of Plant Sciences, Rajarata University of Sri Lanka, Anuradhapura, Sri Lanka; 7 Biodiversity and Palaeobiology (Fungi) Gr., MACS Agharkar Research Institute, Pune, Maharashtra, India; 8 College of Biodiversity Conservation, Southwest Forestry University, Kunming, China

**Keywords:** ammonia fungi, cadaver decomposition island, forensic mycology, fungal succession, post-mortem interval, thanatomycology

## Abstract

Although forensic mycology has primarily focused on microscale Ascomycota and successional patterns, this review emphasizes the underexplored potential of macroscopic Basidiomycota as biomarkers in decomposition ecology. These taxa require quantitative validation prior to application. We present a conceptual synthesis and hypothesis-generating framework, rather than a formal systematic review. We did not utilize pre-registered criteria, formal protocols, or bias assessment. Consequently, conclusions are provisional and intended to encourage empirical validation. Focusing on ammonia fungi and late-stage decomposers, we demonstrate their forensic value through nitrogen-responsive colonization, enzyme-mediated specificity for recalcitrant tissues, and physical persistence of sporocarps and mycelial networks that outlast insect evidence. Unlike transient microbial communities, these fungi form durable, anchored sporocarps detectable in challenging environments where conventional methods fail—including arid regions and cold climates. However, in aquatic systems, Basidiomycota succession is reduced, with bacterial communities offering higher resolution for submersion intervals. Habitat-specific constraints require stratified validation before universal applicability is claimed. Their responsiveness to nitrogenous byproducts generates successional patterns that can assist post-mortem interval estimation. Their enzymatic capacity to degrade resistant materials establishes Basidiomycota as a dominant signature during advanced decomposition. Interactions with insects and grave-soil microbiota produce complex networks yielding additional crime scene evidence. Despite this potential, limitations including inconsistent collection protocols, incomplete databases, and environmental variability hinder broader application. We propose integrating fungal surveys with molecular techniques to develop calibrated forensic models. This synthesis identifies Basidiomycota as a promising biomarker, though evidentiary value requires validation through multi-site empirical studies. With rigorous methodological development, these fungi could serve as transformative tools for clandestine grave detection, crime scene reconstruction, and postmortem interval estimation. This review emphasizes the need for expanded research and standardization to realize the evidentiary value of these underutilized forensic indicators.

## 1 Introduction

Forensic science has traditionally relied on entomology, microbiology, and chemical analysis to estimate post-mortem intervals (PMIs) and study decomposition. Yet, one principal biological player, macroscopic fungi, has received little attention. While insects and bacteria dominate current forensic models, mushrooms, the fruiting bodies of fungi, offer alternative benefits in that they are substrate-dependent, have a predictable succession, and respond to cadaveric decomposition (Sagara, 1975a; Fu et al., 2019). The historical neglect of fungi in forensics stems from several methodological and disciplinary challenges, including the seasonal and ephemeral nature of sporocarps, which contrasts with the more constant availability of insect evidence; a pronounced taxonomic impediment requiring specialist knowledge not typically found in crime labs; and the early focus on culturable, microscopic Ascomycota (Hawksworth and Wiltshire, 2011) or culturing-based methods (Tranchida et al., 2021), which overlooked the ecological significance of macroscopic Basidiomycota.

This review provides a novel and comprehensive synthesis dedicated to establishing macroscopic Basidiomycota as primary forensic biomarkers. We aim to: (1) consolidate Basidiomycota-specific succession patterns in human decomposition, (2) evaluate their enzymatic specialization in late-stage decay, and (3) propose standardized protocols for sporocarp collection and analysis. Recent metabarcoding studies using porcine models reveal environment-dependent fungal colonization patterns, with Ascomycota such as *Yarrowia lipolytica* dominating early outdoor decomposition (Fu et al., 2019), while the forensic utility of Basidiomycota remains underexplored despite their visible sporocarps and nitrogen-responsive ecology.

**Figure 1:**
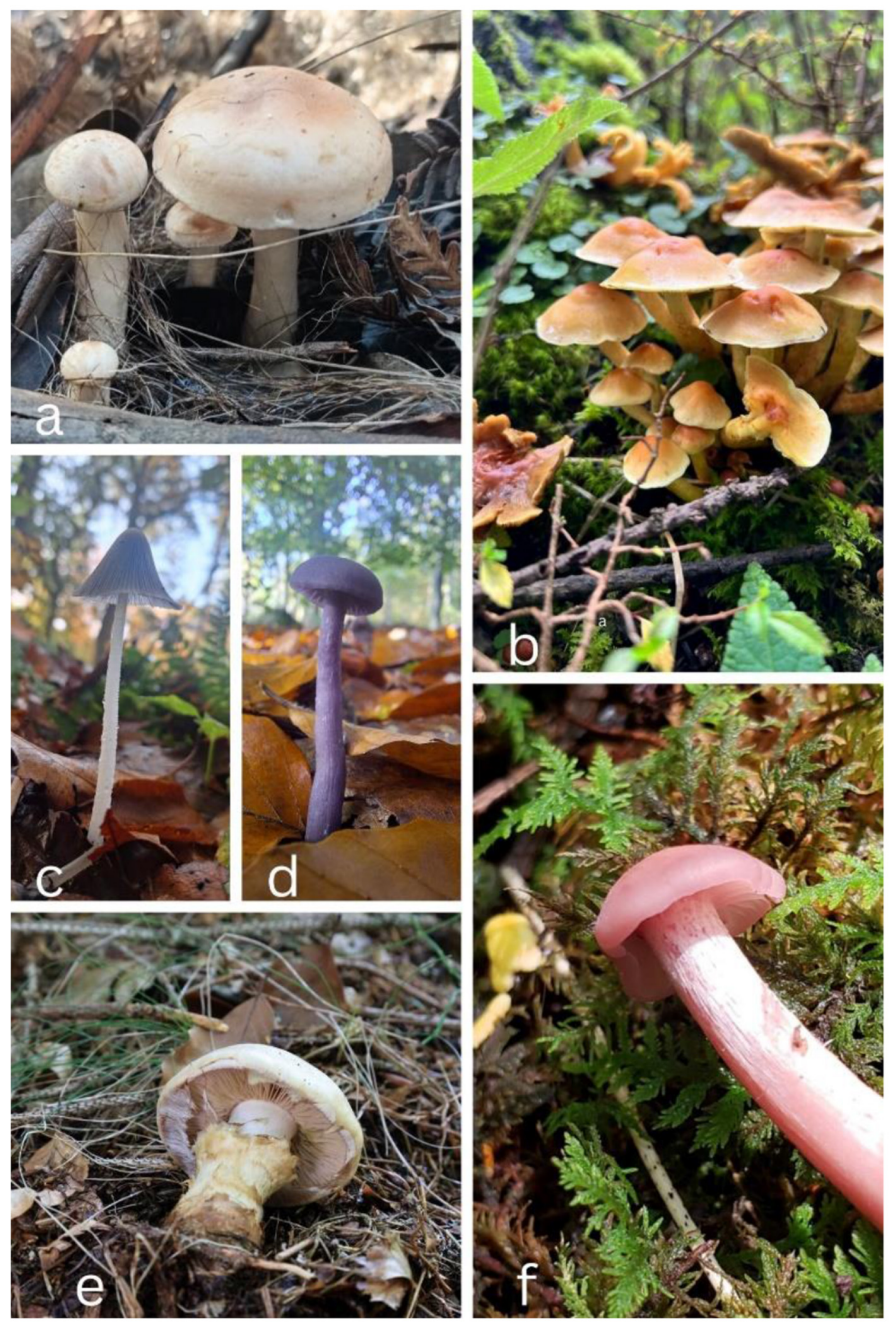
Forensically relevant Basidiomycota species associated with human cadaver decomposition in terrestrial environments, particularly late-stage decomposition and ammonia-rich grave soils. **(a)** *Hebeloma aminophilum*. **(b)** *Hypholoma fasciculare*. **(c)** *Coprinopsis lagopus*. **(d)** *Laccaria amethystina*. **(e)** *Hebeloma radicosum*. **(f)** *Laccaria bicolor*. (https://www.inaturalist.org/, the images are used under the license Attribution Non-Commercial-No Derivs 4.0).

Basidiomycota, particularly ammonia fungi (e.g., *Hebeloma*) and postputrefaction taxa (Figure 1), provide unique forensic advantages as field-detectable decomposition markers. These macrofungi offer multiple applications: estimating PMI when entomological evidence is absent, locating clandestine graves through spore traces, and aiding cases involving mycotoxins (Hawksworth and Wiltshire, 2011).

**Figure 2:**
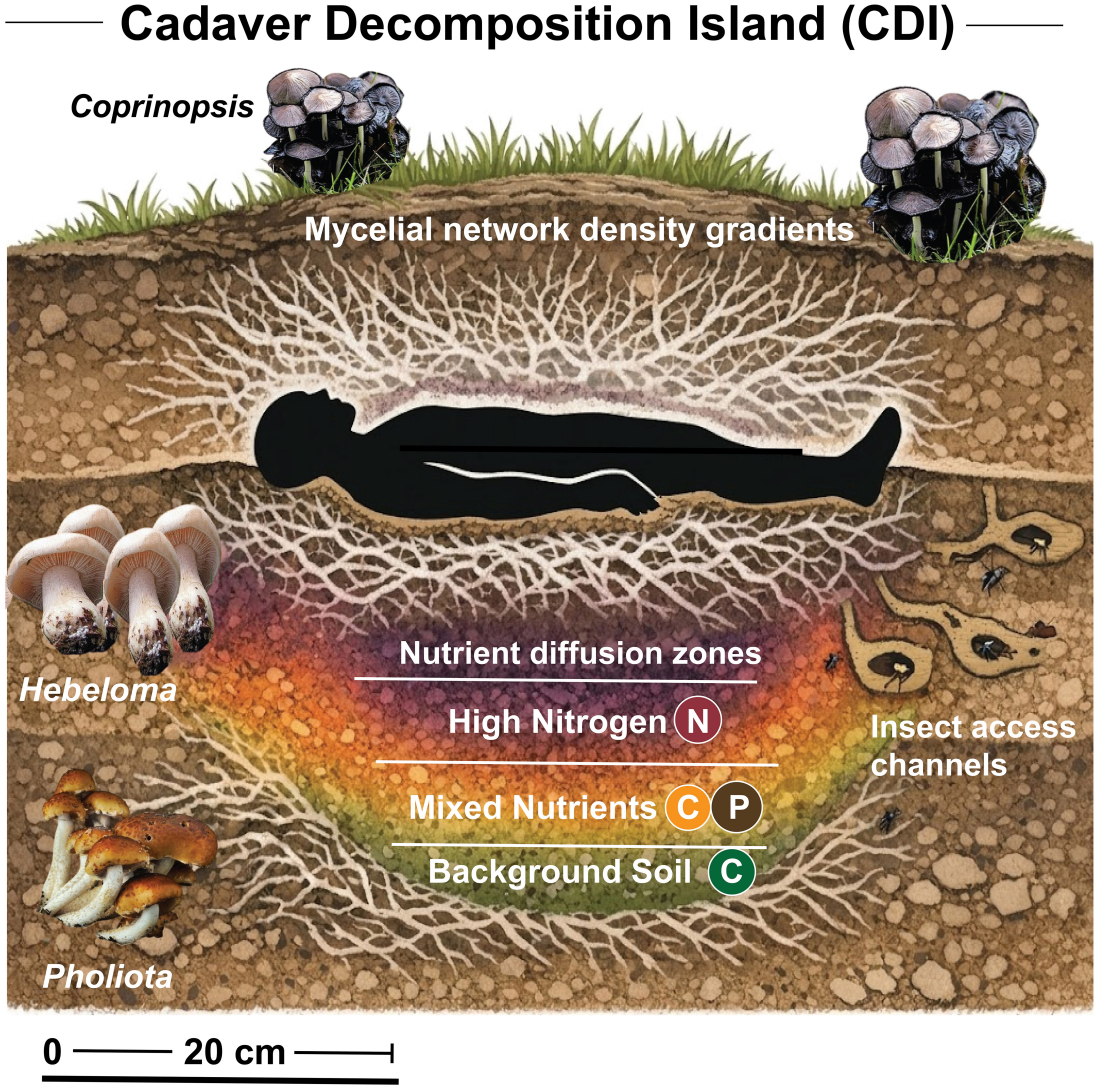
A schematic cross-section of cadaver decomposition island (CDI) dynamics, illustrating three expected forensic relationships pending quantitative validation: (1) vertical stratification of fungal taxa (e.g., deep-penetrating *Hebeloma radicosum* mycelia vs. surface-fruiting *Coprinopsis* spp.), (2) nutrient diffusion gradients from ammonia-rich cores, and (3) insect-fungal interaction zones. This schematic aligns with established observations of ammonia fungi fruiting near remains (Sagara, 1976, 1995) and with fungal depth stratification documented in non-forensic soils. The spatial relationships depicted are conceptual; validation of stratification patterns and diffusion zones across environments requires depth-resolved ITS1 metabarcoding and soil chemistry profiling (ammonium, pH). The conceptual diagram was created with AI assistance. Supporting citations and evidence are provided in the main text. This figure is intended to generate testable hypotheses rather than to present quantitative empirical data.

Although Figure 2 presents a conceptual model of spatially organized fungal networks within CDIs, quantitative evidence directly linking ITS-sequenced fungal communities to measured ammonium and pH gradients in vertical transects is currently unavailable. Bacterial spatial profiling using nutrient assays is well established in grave soils (Carter et al., 2007), and fungal depth stratification has been documented in non-forensic soils (Baldrian, 2006). However, no study has yet integrated these methodologies within actual CDIs. The relationships illustrated in Figure 2 are therefore hypothesized based on extrapolation from ammonia fungus fruiting patterns (Sagara, 1976, 1995) and general soil ecology. Empirical validation through depth-resolved ITS metabarcoding in conjunction with soil chemistry profiling remains a critical research priority.

**Figure 3:**
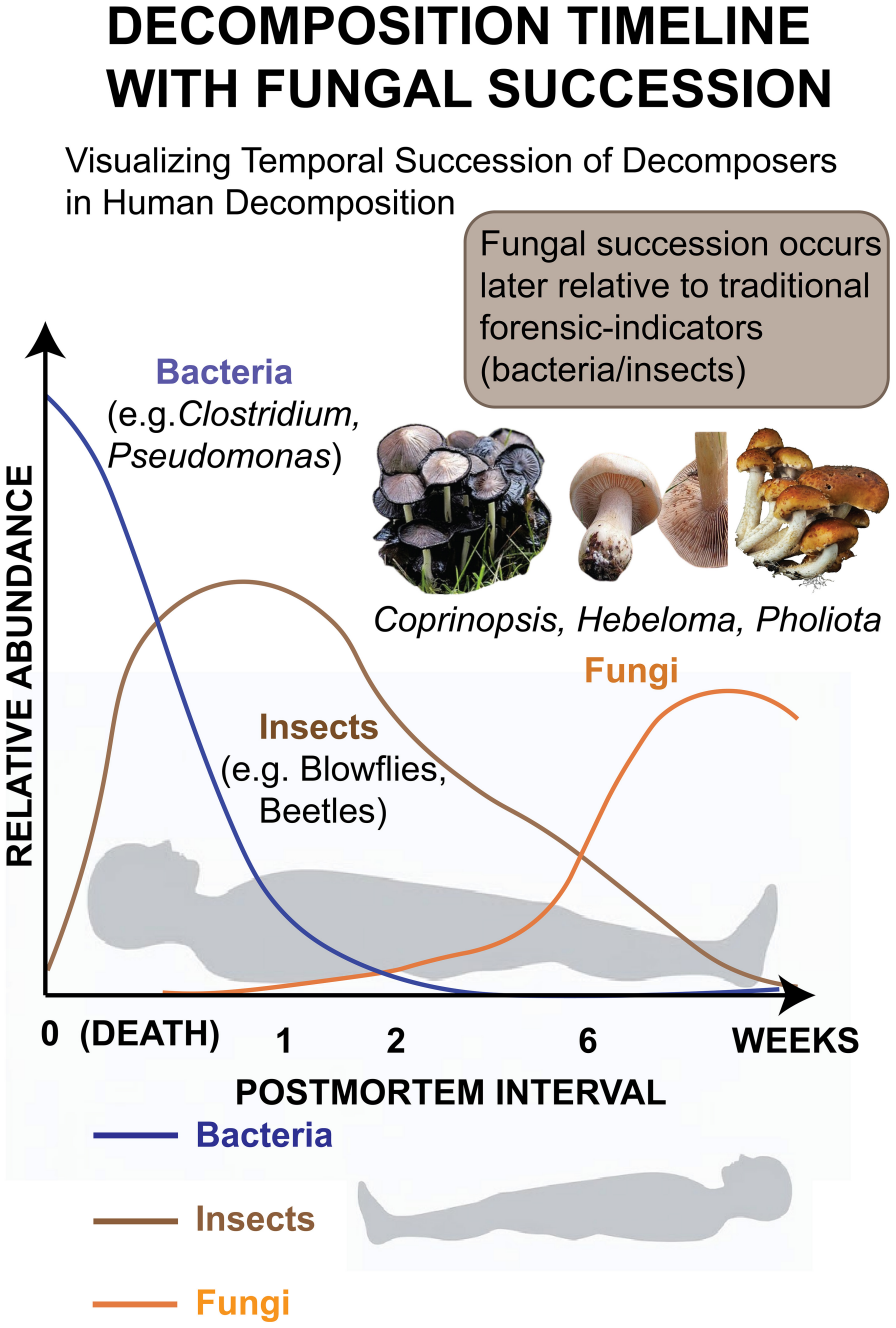
Temporal succession of decomposers during human decomposition, illustrating the relative abundance of bacteria, insects, and fungi with particular emphasis on mushroom-forming genera over time. Illustrative conceptual diagram created with AI assistance; see text for supporting citations and evidence. This figure is intended to generate testable hypotheses, not to present quantitative empirical data.

Unlike microbial succession models that require invasive sampling, macrofungi such as *Hebeloma vinosophyllum* provide non-destructive, surface-accessible indicators that respond persistently to nitrogenous compounds such as cadaverine (Hibbett and Donoghue, 2001; Deel et al., 2021). Fungal succession patterns complement existing forensic tools (Figure 3). Insect succession on cadavers follows predictable patterns: blowflies (Calliphoridae) typically colonize within minutes to hours post-mortem (fresh stage), followed by beetles (Silphidae, Staphylinidae) during active decay. Later-stage colonizers, such as Piophilidae and Dermestidae, appear during advanced decay and dry remains (Amendt et al., 2004). These entomological timelines serve as the comparative framework for calibrating fungal succession patterns.

*Coprinopsis* species thrive in nitrogen-rich conditions by metabolizing putrescine and cadaverine (Stamets, 2005), while *Hebeloma* and *Pholiota* sporocarps fruit predictably from grave soils (Fu et al., 2019). Unlike insect succession, fungal phenology directly reflects nitrogen fluxes during putrefaction, providing biochemical timelines for PMI estimation (Tibbett and Carter, 2003). In burial contexts, oxygen limitation shifts dominance toward Ascomycota, such as Pyronemataceae and *Talaromyces udagawae* (Procopio et al., 2020; Tranchida and Cabello, 2017), though Basidiomycota remain essential surface indicators due to their enzymatic specialization exemplified by *Pleurotus pulmonarius*, which degrades α-keratin in human hair (Inácio et al., 2017).

Although environmental factors influence forensic patterns, they do not obscure the core signatures. Decomposer taxa such as *Mortierella* and *Coprinopsis* are consistently observed across diverse ecosystems. Microbial community profiles were traditionally clustered into Operational Taxonomic Units (OTUs) based on 97% sequence similarity in earlier studies (Metcalf et al., 2014, 2016). More recently, Amplicon Sequence Variants (ASVs) have provided single-nucleotide resolution in modern metabarcoding, surpassing soil type in postmortem interval (PMI) prediction (Callahan et al., 2017). ITS2 metabarcoding enables effective detection of rare taxa (Procopio et al., 2020), and fungal volatile organic compounds (VOCs) facilitate cadaver dog detection (Pacioni et al., 2015). However, forensic mycology continues to face challenges, including inconsistent protocols and taxonomic gaps (Tranchida et al., 2021). This review synthesizes current knowledge to advance Basidiomycota as indispensable forensic indicators when conventional methods fail.

### 1.1 Scope and methodological approach

The methodological scope of this review is clarified as follows. This work provides a conceptual synthesis that integrates foundational observational studies in ammonia fungus ecology, primarily the pioneering research of Sagara (1973, 1975a,b, 1976, 1981, 1989, 1992, 1995); (Sagara et al. 1981, 1985, 1993a,b, 2000, 2008), with recent molecular investigations. It does not constitute a formal systematic review or meta-analysis; PRISMA guidelines, registered search protocols, inclusion and exclusion criteria, and quantitative bias assessment were not employed. As a result, there is potential for selection bias toward studies that support the central thesis, a recognized limitation of narrative reviews (Gurevitch et al., 2018; Koricheva et al., 2013). The figures included are conceptual integrations intended to generate testable hypotheses rather than quantitative data summaries. Validation through standardized, multi-site empirical studies with formal evidence synthesis is necessary before forensic application.

## 2 The ecology of fungal decomposition

### 2.1 Role of macrofungi in cadaver decomposition

**Figure 4:**
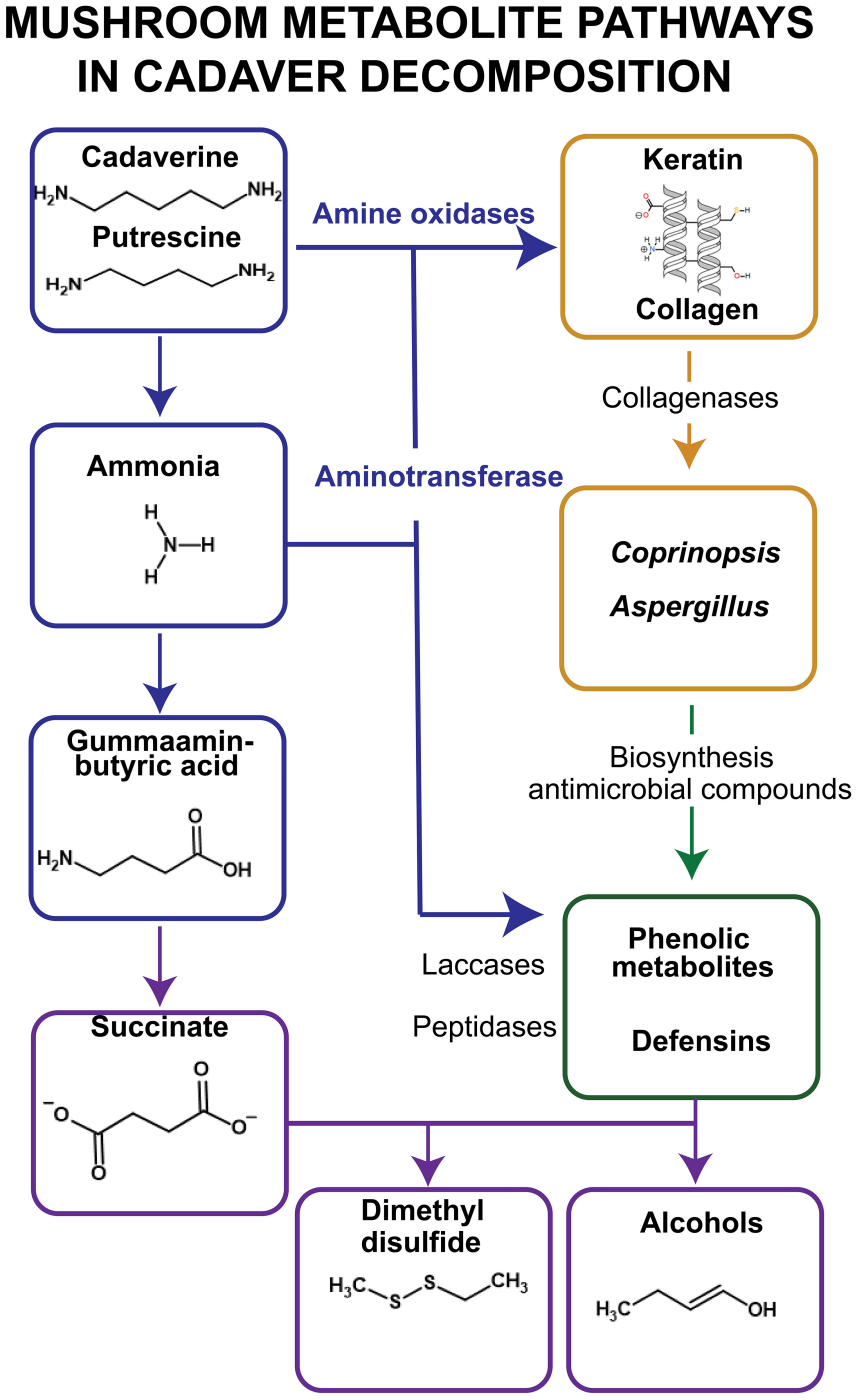
A conceptual summary of candidate biochemical pathways involved in mushroom-mediated decomposition processes. Illustrative conceptual diagram created with AI assistance; see text for supporting citations and evidence. This figure is intended to generate testable hypotheses, not to present quantitative empirical data. These pathways are conceptually derived from documented fungal enzyme repertoires identified in non-forensic contexts such as soil decomposition and industrial applications. However, taxon-resolved functional meta-omics data (genomics, transcriptomics, proteomics, metabolomics) from cadaver decomposition island (CDI) soils were not available at the time of this review. Consequently, attributing these pathways to specific Basidiomycota taxa in forensic contexts remains hypothetical and requires empirical validation. The flowchart presents potential mechanisms, including the degradation of cadaverine and putrescine by amine oxidases, the enzymatic breakdown of keratin and collagen by keratinases and collagenases, the synthesis of antimicrobial compounds, and the production of volatile organic compounds such as geosmin. This figure summarizes candidate mechanisms that await taxon-resolved validation in CDI contexts.

Whereas bacteria have been the traditional target of forensic decomposition studies, macrofungi, and specifically mushrooms, are a vital but underrated vertebrate degrader. Fruiting bodies produced by Ascomycota and Basidiomycota are visible signs of fungal invasion and possess the biochemical capacity to dissolve complicated biopolymers such as keratin, collagen, and lipids through special enzymatic processes (Hibbett and Donoghue, 2001). Although Basidiomycota enzyme capabilities, such as keratinases, collagenases, and amine oxidases, are well documented in non-cadaver decomposition island (CDI) soils and industrial contexts (Baldrian, 2006; Floudas et al., 2012), their specific activity within CDIs has not been directly demonstrated using functional meta-omics. The pathways discussed below and summarized in Figure 4 are conceptually derived from established fungal enzyme repertoires and constitute testable hypotheses that require empirical validation in forensic contexts.

Fu et al. (2019) observed more evident mildew blooms with *Yarrowia lipolytica* on pig carcasses during outdoor decomposition, particularly under humid conditions. These are Ascomycota activity, and Basidiomycota like *H. vinosophyllum* exhibit an analogous forensic use by forming sporocarps through nitrogen fluxes at tertiary-stage decomposition (Sagara, 1995). Fungi such as *Coprinopsis, Psilocybe*, and *Pholiota* tend to inhabit the vicinity of carcasses, where they participate in nutrient cycling and are usually dominant over bacteria in nitrogen-rich soil due to their advanced lignocellulose-degrading machinery (Boddy and Jones, 2008). Longitudinal persistence benchmarks that compare sporocarp survival windows to insect and bacterial evidence have not yet been established. Although qualitative observations indicate that sporocarps may persist for 3 to 8 months in certain species (e.g., *H. vinosophyllum*), systematic survival curves or detection-window comparisons across biomes are lacking. The literature variably characterizes sporocarp duration as short-lived (*Coprinopsis* spp., 24 to 48 hours) or persistent (*Hebeloma* spp.), but does not provide standardized cadaver decomposition index (CDI) benchmarks for comparison with insect or bacterial evidence persistence.

Formation of mushrooms over decaying corpses follows temporally predictable patterns of decay chemistry. Early decomposition, regulated by anaerobic bacteria such as *Penicillium* and *Aspergillus* (Hitosugi et al., 2006; Ishii et al., 2006; Schwarz et al., 2015), typically excludes Basidiomycota. As oxygen levels increase and putrefactive volatiles decrease, necrotrophic fungi that thrive in protein-rich environments become dominant. *H. vinosophyllum*, for instance, responds specifically to elevated nitrogen content at advanced decay (Sagara, 1976; Sagara et al., 1993a). However, quantitative ammonium thresholds for fruiting initiation under CDI-like conditions remain to be established. No dose-response data currently define the nitrogen concentrations required to trigger sporocarp formation, nor has fruiting reproducibility been tested across varying environmental covariates such as pH, moisture, and temperature. Key research priorities must include controlled mesocosm experiments using urea-amended soils to track fruiting in response to nitrogen flux while systematically varying these confounders (Stamets, 2005). Despite forensic reliance on certain Ascomycota, aquatic work by Hawksworth and Wiltshire (2011) showed that the development of hyphal sheaths follows regular timelines on submerged remains, a terrestrial model for Basidiomycota succession. For this field to advance, a critical priority is the establishment of long-term, regionally calibrated studies across major biomes. We propose a coordinated global effort, using standardized vertebrate models and molecular techniques, to document how fungal colonization patterns are modulated by climate, soil type, and season. Key research priorities must include the controlled mesocosm experiments to isolate the effects of humidity and temperature on sporocarp formation in key forensic species like *Hebeloma*; (ii) the use of defined cadaver analogs (e.g., urea-amended soils) to precisely track fruiting body initiation in response to nitrogen flux; and (iii) the creation of curated, public databases for fungal-cadaver interactions (Stamets, 2005).

Experiments on *Sus scrofa* cadavers have confirmed their supremacy in cadaver decomposition islands (CDIs), although 18S rRNA data did not statistically distinguish from bacterial communities (Figure 3; Chimutsa et al., 2015). This means that fungal indicators, although evident, may require longer observation periods or integration with molecular data. Fungal colonization appears to be influenced by environmental factors such as moisture, temperature, and burial depth. For instance, superficial burials prefer species such as *Mycena* and *Gymnopus*, which prefer shaded, cooler conditions (Sagara, 1995). On the contrary, xerotolerant fungi species like *Panaeolus* prefer exposed surface remains. Such ecological preferences enhance the utility of mushrooms as bioindicators in estimating postmortem intervals (PMI) and depicting burial conditions. Mushrooms have numerous distinct forensic applications. Certain species, such as *Pholiota highlandensis*, grow almost exclusively on animal tissue, making them useful for detecting cadavers (Sagara et al., 2008). Fungal mycelium in soil is also utilized to detect concealed graves after skeletonization (Anderson et al., 2013). Despite this potential, the lack of standardized protocols for correlating fungal appearance and decomposition stages has hindered operational application. Local accounts of succession patterns across climates and burial environments are required for practical field application. Recent research integrates machine learning with fungal succession data to improve the accuracy of PMI estimation.

In a Tennessee study with 19 human donors, ITS-based models using fungi were initially poorer than bacterial 16S models, but became much more predictive upon the addition of environmental factors such as soil conductivity and pH (Mason et al., 2024). This kind of outcome aligns with the postulation that nitrogen-responsive Basidiomycota fruiting requires contextual environmental information for proper modeling (Sagara, 1995). In a Swiss murder case, radiocarbon dating provided a PMI of 1–3 years, and sequencing of microeukaryotes in soil beneath the body revealed spatially distinct microbial communities, consistent with body-zone patterns and *Hebeloma radicosum* CDI development (Szelecz et al., 2018). This case emphasized three forensic uses: fine-tuning rough PMI estimates through sporocarp succession, outlining decomposition zones by mycelial expansion, and the possible application of spores adhering to substrates as trace evidence connecting deposition points. Molecular research is also transforming forensic mycology.

ITS high-throughput sequencing of decomposing rodents revealed time-specific fungal shifts, in which *Aspergillus* and *Ophiocordyceps* exhibit stage-specific relative abundances. Such studies, however, tend to focus on early-decay Ascomycota rather than ecologically significant Basidiomycota. Coupling sporocarp surveys with high-throughput sequencing will better record the entire fungal succession. Tools like the “ITS Metagenomics” platform (Giampaoli et al., 2021) show that cloud-based, standardized pipelines can achieve high genus-level accuracy and render data comparability among studies higher. Such a methodology, when allied with databases like UNITE, could potentially construct an integrated thanatomycological framework of forensic practice. Environmental heterogeneity significantly influences fungal succession. In the Brazilian savannah biome, *Sus scrofa* cadavers showed *Aspergillus terreus* and Mucorales predominating in active decay, with scanty Basidiomycota (Leite-Jr et al., 2019). This contrasts with temperate habitats, where nitrogen-fixing Basidiomycetes such as *Hebeloma* and *Coprinopsis* are always present with late decomposition. Adult remains, such as the Kuffner family mummies, contain distinctive Ascomycota-dominated communities that succeed in desiccated tissue, dominated by *Penicillium* and *Aspergillus* (Šimonovičová et al., 2015). The strains were very cellulolytic and keratinolytic on skin and fabrics, with airborne isolates exerting greater destructive potential than endogenous ones. This movement is countered by the nitrogen-dependent Basidiomycota ecology found in fresh remains, which affirms the significance of the decomposition environment and state in interpreting fungal evidence. Tables 1, 2 list forensically important Basidiomycota species that are often associated with human decomposition, and presumptively forensic yeast-like and mycelial Basidiomycetes, respectively.

**Table 1:** Forensically relevant Basidiomycota associated with decomposition.

Taxon	Order	Family	Geographic distribution	Forensic utility	References
*Coprinopsis echinosporus*	Agaricales	Psathyrellaceae	Temperate grasslands (Japan)	Short-lived sporocarps indicate recent cadaveric nitrogen spikes; PMI estimation	Sagara, 1973, 1975a, 1992
*C. neolagopus*			Temperate forests	Emerges quickly postputrefaction; useful in recent burial detection	Sagara, 1973, 1975a, 1992, 1995
*C. phlyctidosporus*			Temperate woodlands	Indicator of high-nitrogen late decomposition soils	Sagara, 1973, 1975a, 1992, 1995
*Hebeloma aminophilum*		Hymenogastraceae	Australia (temperate)	Strong ammonia fungus; fruits near remains; grave locator	Hilton, 1978; Miller and Hilton, 1986
*H. luchuense*			Subtropical Asia (Japan, Ryukyu)	Ammonia fungus; habitat-limited fruiting near remains	Fukiharu and Hongo, 1995
*H. radicosoides*			Japan (montane soils)	Indicator of subterranean remains; roots through burial layers	Sagara, 1973, 1975a, 1992, 1995; Kuroyanagi et al., 1982
*H. radicosum*			Temperate Europe and Asia	Diagnostic sporocarp for buried cadavers in advanced decay	Kuroyanagi et al., 1982; 1995
*H. spoliatum*			Temperate deciduous forests	Persistently fruits near nitrogen-rich grave soils	Fukiharu et al., 2000a; Sagara, 1992, 1995
*H. vinosophyllum*			Japan, Korea	Reliable ammonia fungus; potential for long-term grave detection	Fukiharu et al., 2000a,b, Sagara, 1992, 1995
*Hypholoma fasciculare*		Strophariaceae	Cosmopolitan	Late-stage decomposer; visible, persistent clusters on wood/cadavers	Piepenbring et al., 2025
*Laccaria bicolor*		Hydnangiaceae	Northern Hemisphere forests	Ectomycorrhizal; occasionally near decomposed remains in root zones	Sagara, 1973, 1975a,b, 1976, 1981, 1989, 1992, 1995; Sagara et al., 1981, 1985, 1993a,b, 2000, 2008
*L. amethystina*			Europe, Asia, North America	Not typically cadaveric but may fruit in disturbed forest soils	Sagara, 1995
*Laccaria* sp.			Global	Opportunistic colonizer of grave soils under trees	Sagara, 1995
*Lactarius chrysorrheus*	Russulales	Russulaceae	Europe, N. Africa	Appears in nitrogen-altered woodlands; secondary grave marker	Sagara, 1973, 1975a,b, 1976, 1981, 1989, 1992, 1995; Sagara et al., 1981, 1985, 1993a,b, 2000, 2008
*Lepista nuda*	Agaricales	Tricholomataceae	Temperate zones	May grow in cadaveric nutrient-enriched soils; useful in long-term scenes	Sagara, 1995
*Panaeolina sagarae*		Bolbitiaceae	Japan	Forensically rare; appears in nutrient-rich, disturbed sites	Sagara, 1973, 1975a,b, 1976, 1981, 1989, 1992, 1995; Sagara et al., 1981, 1985, 1993a,b, 2000, 2008
*Tephrocybe ambusta*		Lyophyllaceae	European woodlands	Postputrefaction; moderate nitrogen preference; late-stage indicator	Sagara, 1973, 1975a,b, 1976, 1981, 1989, 1992, 1995; Sagara et al., 1981, 1985, 1993a,b, 2000, 2008
*T. tesquorum*			Arid to semi-arid regions	May indicate decomposition in dry soils or shallow burials	Sagara, 1973, 1975a,b, 1976, 1981, 1989, 1992, 1995; Sagara et al., 1993b,a, 1981, 1985, 2000, 2008
*Rhizopogon succosus*	Boletales	Rhizopogonaceae	Pine forests	Hypogeous sporocarps may mark long-buried remains; limited visibility	Sagara, 1973, 1975a,b, 1976, 1981, 1989, 1992, 1995; Sagara et al., 1981, 1985, 1993a,b, 2000, 2008
*Suillus luteus*		Suillaceae	Northern Hemisphere (pine)	May occur near roots of decomposing trees near remains	Sagara, 1995
*S. bovinus*			Temperate pine forests	Indirect indicator of forest cadaver disturbance	Sagara, 1995

**Table 2:** Putatively forensic yeast-like and mycelial basidiomycetes.

Taxon	Order	Family	Geographic Distribution	Forensic Utility	References
*Cryptococcus* sp.	Tremellales	Tremellaceae	Cosmopolitan	Frequently isolated from internal tissues; forensic potential in cold cases	Tranchida and Cabello, 2017; Kaszubinski et al., 2022
*Haplotrichum* sp.	Cantharellales	Incertae sedis	Unknown	Early colonizer of cadaveric skin; forensic relevance unclear	Hawksworth and Wiltshire, 2011; Tranchida et al., 2021
*Rhizoctonia* sp.	Cantharellales	Ceratobasidiaceae	Global (soil-associated)	Root-zone fungus; may indicate disturbed burial sites	Carter and Tibbett, 2008; Hawksworth and Wiltshire, 2011
*Rhodotorula* sp.	Sporidiobolales	Sporidiobolaceae	Global (skin, air, soil)	Pigmented yeast; sometimes isolated from cadaveric environments	Sidrim et al., 2010; Procopio et al., 2020
*Trichosporon* sp.	Trichosporonales	Trichosporonaceae	Widespread	Found on nails, hair, and skin; relevant in late-stage remains	Inácio et al., 2017; Spychała et al., 2024

Taxonomic verification for Tables 1 and 2: Species names and current taxonomy were verified against Index Fungorum (www.indexfungorum.org) and MycoBank (www.mycobank.org) databases as of March 2026. Higher-level classification follows recent phylogenetic classifications.

### 2.2 Mushrooms of forensic importance in decomposition

The fungal kingdom encompasses a wide variety of macroscopic species whose ecological roles intersect significantly with the process of cadaver decomposition (Figure 4). Among these organisms, certain mushrooms demonstrate observational associations with decaying remains, indicating potential forensic applications. However, these associations are currently supported by correlational data rather than quantitative predictive models. The following sections discuss taxa that appear promising as forensic indicators, while noting that their nitrogen-responsive ecology necessitates quantitative threshold validation and that their persistence advantages require systematic comparison with insect and bacterial evidence. These predictable associations are spatially organized within distinct zones of the Cadaver Decomposition Island (CDI). Figure 3 presents a cross-sectional model of CDI dynamics, highlighting three critical forensic relationships: (1) vertical stratification of fungal taxa (e.g., *H. radicosum* mycelia penetrating burial layers versus surface-fruiting *Coprinopsis* spp.); (2) nutrient diffusion gradients driving succession (ammonia-rich zones attracting nitrogen-responsive fungi); and (3) insect-fungal interaction hotspots where mycophagous arthropods disperse spores. This spatial framework underpins the taxonomic specialization discussed below.

#### 2.2.1 Primary decomposer mushrooms (saprotrophs)

Primary saprotrophic decomposers are the first macroscopic fungi to directly infect cadaveric tissue, initiating the degradation of complex polymers such as cellulose, lignin, and chitin. These early-stage fungi, represented by the genera *Coprinopsis* and *Hypholoma*, are ecologically distinct from the following necrotrophs in that they target the structural components of the decomposing corpse and its immediate environment, such as submersed wood or leaf litter, and decompose them with fluid saturation. Their rapid response to nutrient influx renders them excellent early warning indicators of a CDI. To illustrate, *Coprinopsis* species are rapid to fruit on nitrogen-supplemented soil, their short-lived sporocarps providing an unambiguous, albeit fleeting, sign of recent decomposition activity.

#### 2.2.2 Necrotrophic mushrooms

Recent advances in forensic mycology have revealed the exceptional evidentiary value of necrotrophic Basidiomycota, particularly species in the genus *Hebeloma*, due to their predictable fruiting patterns above buried cadavers. This phenomenon, extensively documented by Fu et al. (2019) and Vidona et al. (2025), represents a paradigm shift in grave detection methodologies. Unlike traditional Ascomycota-based approaches requiring laboratory culturing, these visible sporocarp-forming fungi, such as *Hebeloma syrjense* and *H*. *vinosophyllum*, demonstrate remarkable consistency in CDIs. The latter species typically produces sporocarps 3–8 months post-burial in nitrogen-enriched grave soils, serving as both spatial markers for grave localization and temporal indicators of advanced decomposition stages.

The forensic application of these necrotrophic specialists extends beyond *Hebeloma* to include other Basidiomycota exhibiting distinct decomposition signatures. *Coprinopsis atramentaria* and *C*. *radiata*, for instance, respond rapidly to nitrogenous compounds like putrescine, emerging on necrotic tissue with diagnostically precise but ephemeral sporocarps that persist for only 24–48 h (Stamets, 2005). This brief yet predictable fruiting window provides investigators with critical temporal resolution for estimating periods of active decomposition. Similarly, Pholiota highlandensis demonstrates near-exclusive colonization of mammalian cadavers, establishing its value as a highly specific biomarker for the localization of human remains (Sagara et al., 2008). These necrotrophic fungi are intrinsically linked to postmortem biochemical changes, with certain species functioning as sensitive chemical indicators through their fruiting responses to decomposition-driven alterations in soil chemistry. Figure 4 summarizes candidate mechanisms and volatiles that require taxon-resolved validation in CDI contexts. Although the enzymatic capabilities illustrated, such as amine oxidases, keratinases, and collagenases, are well documented in Basidiomycota from non-forensic soils and industrial applications (Baldrian, 2006; Floudas et al., 2012), their specific activity within CDIs and their assignment to particular fungal taxa have not been directly demonstrated using functional meta-omics. The production of volatiles, including geosmin, by forensic fungi (Pacioni et al., 2015) presents promising opportunities for cadaver dog training and volatile organic compound (VOC) profiling. However, quantitative flux measurements and taxon-resolved activity data from CDI contexts are still required to substantiate these applications.

Normally mycorrhizal, *Entoloma rhodopolium* has been found to fruit prolifically in the vicinity of residues in response to high ammonia concentrations from protein decomposition (Hawksworth and Wiltshire, 2015). Likewise, certain *Psilocybe* spp., such as the hallucinogenic *P*. *semilanceata*, show heightened fruiting in cadaveric soil, presumably in response to localized changes in micronutrient availability (Anderson et al., 2013). They supplement necrotrophic fungi by providing indirect evidence of decay through their biochemical responses, even when sporocarps are not directly associated with remains. The forensic investigation of such fungal patterns requires understanding their complex interactions with other decomposer assemblages. Even though nitrogen-responsive Basidiomycota prevail in surface decomposition, molecular analyses indicate that skeletal remains harbor unique microbial communities, led by Proteobacteria (36%) and Actinobacteria (23%), and supplemented by collagenolytic *Clostridium* as human DNA breaks down (Emmons et al., 2020). Such ecological partitioning provides complementary forensic approaches: surface-fungus growth indicates recent decomposition activity, and subsurface bacterial populations track extensive skeletal breakdown.

However, functional application must accommodate high ecological heterogeneity. Studies in Buenos Aires Province revealed the predominance of ascomycetous fungi *Dichotomomyces cejpii* and *Talaromyces udagawae*, in buried remains (Tranchida and Cabello, 2017), and three key species-rich situations in which Ascomycota predominate: anaerobic burial environment, early to middle-phase decomposition (PMI < 6 months), and alkaline soil habit. The stratification emphasizes context-specificity in forensic thanatomycology, with Basidiomycota acting as ideal indicators of surface decomposition during later stages and certain Ascomycota being better suited to buried or unexpected decomposition cases. Having both groups of fungi, along with their patterns of chemical response, provides a robust multi-indicator system for forensic examination.

#### 2.2.3 Opportunistic pathogenic mushrooms

In a few rare decomposition environments, a few mushrooms seem to emerge not only through environmental colonization but also through opportunistic infections that began intra vitam. The wood-decay Basidiomycete *Schizophyllum commune* has been reported to infect immunocompromised patients antemortem and subsequently develop in lung tissue after death, causing visible mycological colonization on forensic examination (Xue et al., 2025). Equally, *Aspergillus fumigatus*, typically microscopic, may form macroscopic fruiting bodies under specific conditions, particularly in hospital settings or when delayed diagnosis occurs. Such cases blur the distinction between environmental fungi and true cadaveric colonizers and require precise differentiation in forensic examination.

Differentiating cadaver-associated mushrooms from environmental saprotrophs relies on several forensic traits, including substrate preference, fruiting sequence, and biochemical profiles. For example, genera such as *Pholiota highlandensis* show a strong preference for putrid animal tissue, whereas soil-inhabiting fungi show no such preference. The time of fruit body appearance, typically at active or advanced rot stages, can also aid postmortem interval estimation. In addition, metabolite profiling detects distinctive volatile organic compounds (VOCs), such as those derived from cadaverine and putrescine metabolism, that are not typically produced by environmental organisms. Physical defects of fungal sporophores, such as geotropic changes in Basidiomycota, have, in some cases, indicated substrate disturbance or body movement at crime scenes (Tranchida et al., 2021), another forensic implication of fungal analysis.

Ongoing research programmes are constructing DNA barcoding libraries for forensic mushrooms, enabling rapid and precise identification of fruiting bodies and spores in decomposition sites. These facilities, coupled with growing ecological data on fungal succession, are rendering mushrooms effective forensic indicators, similar to blowflies and beetles. Their utilization extends even to the examination of soft tissues. Fungi can infest exposed skeletal remains under favorable ecological conditions. Hawksworth and Wiltshire (2011) cultured 13 taxa of fungi from scavenged bones with remaining soft tissue, like Mucorales species that colonized dermal surfaces within 1–2 days post-mortem. Such bone-surface fungi are very important leads in skeletonized cases, especially when traditional forensic markers have decomposed.

Mushrooms are used in forensic science beyond the estimation of the postmortem interval. Mushroom spores find broader forensic applications. Spores in palynological samples have been used as trace evidence to link suspects to graves by identifying genera such as *Coprinopsis* and *Hebeloma*. Fungal spores assisted in verifying nearness to crime scenes in reported criminal cases (Hawksworth and Wiltshire, 2011; Jasim, 2021). Fungal analysis is also utilized in toxicological investigations. Ingestion of psychoactive or poisonous species, such as *Amanita* or *Psilocybe*, can be confirmed through gut content analysis, as demonstrated in a study involving *Psilocybe* spores that led to the conviction of a suspect in a drug homicide case (Jasim, 2021). The majority of cadaver-related Basidiomycota, however, such as *Hebeloma* species documented in temperate Asia and Europe, are not typically poisonous to humans and thus do not influence postmortem toxicology tests (Sagara, 1995; Hawksworth and Wiltshire, 2011). However, regional variation in toxicity should be considered, and consultation with local mycological references is recommended when interpreting toxicological findings in cases involving possible fungal ingestion. Furthermore, forensic mycology aids in identifying illegal activities of mass cultivation of controlled or hallucinogenic fungi, and helps to supply significant evidence for drug cases.

**Figure 5:**
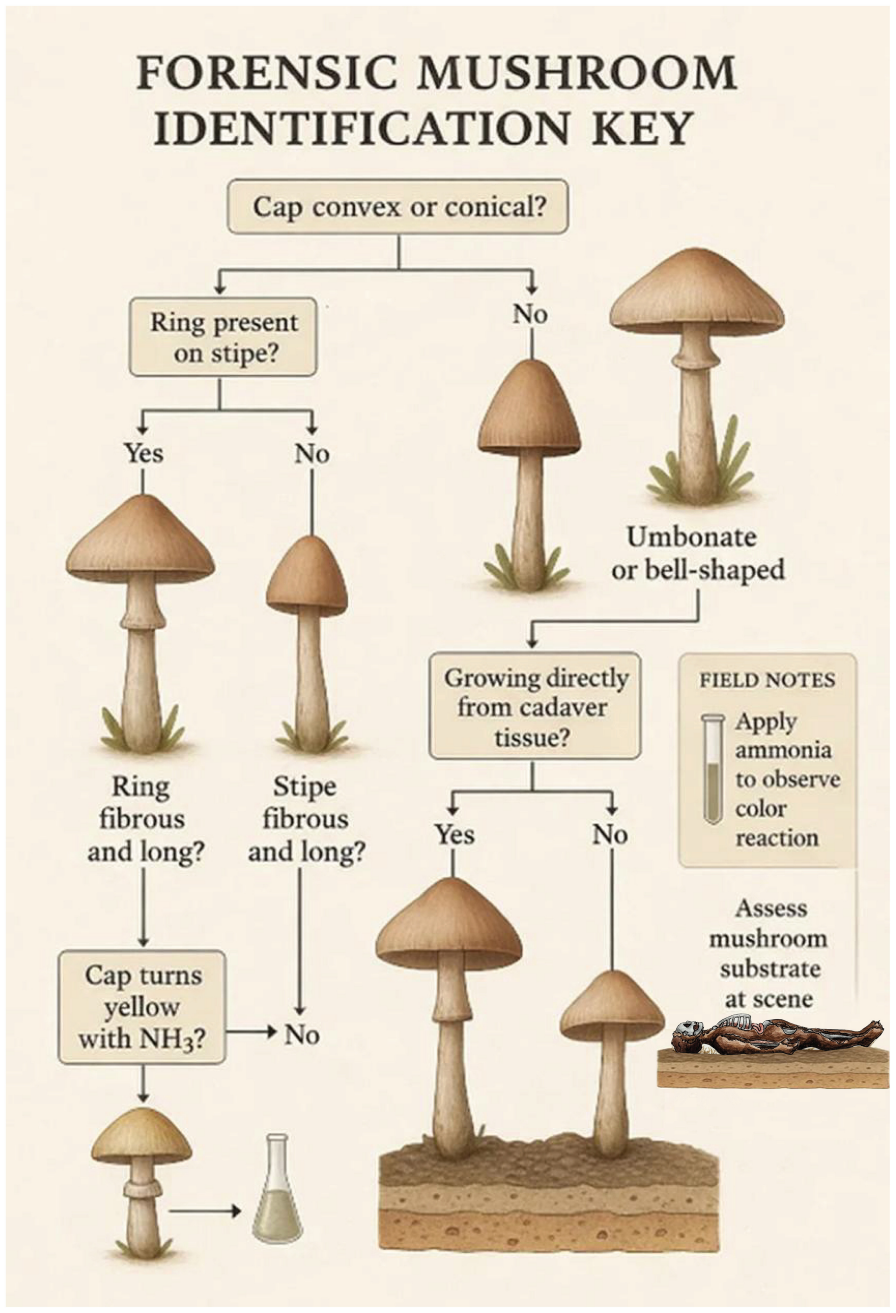
Dichotomous identification key for forensic mushroom assessment, illustrating diagnostic features. Illustrative conceptual diagram created with AI assistance; see text for supporting citations and evidence. This figure is intended to generate testable hypotheses, not to present quantitative empirical data.

Moreover, accurate taxonomic identification remains at the heart of forensic mycological application. Figure 5 illustrates a dichotomous key specifically designed for field identification of mushrooms of forensic worth, emphasizing: macromorphological attributes of sporocarps (cap texture, gill attachment) most stable in decomposition environments; chemical staining reactions typical of cadaver-associated species (e.g., vinaceous bruising of *H. vinosophyllum*); and habitat filters employed to sift out necrophilous fungi from analogous saprotrophs. This empirical method addresses the taxonomic challenges outlined in Section 4, along with the molecular strategies discussed in the future directions.

### 2.3 Interactions with other decomposers

**Figure 6:**
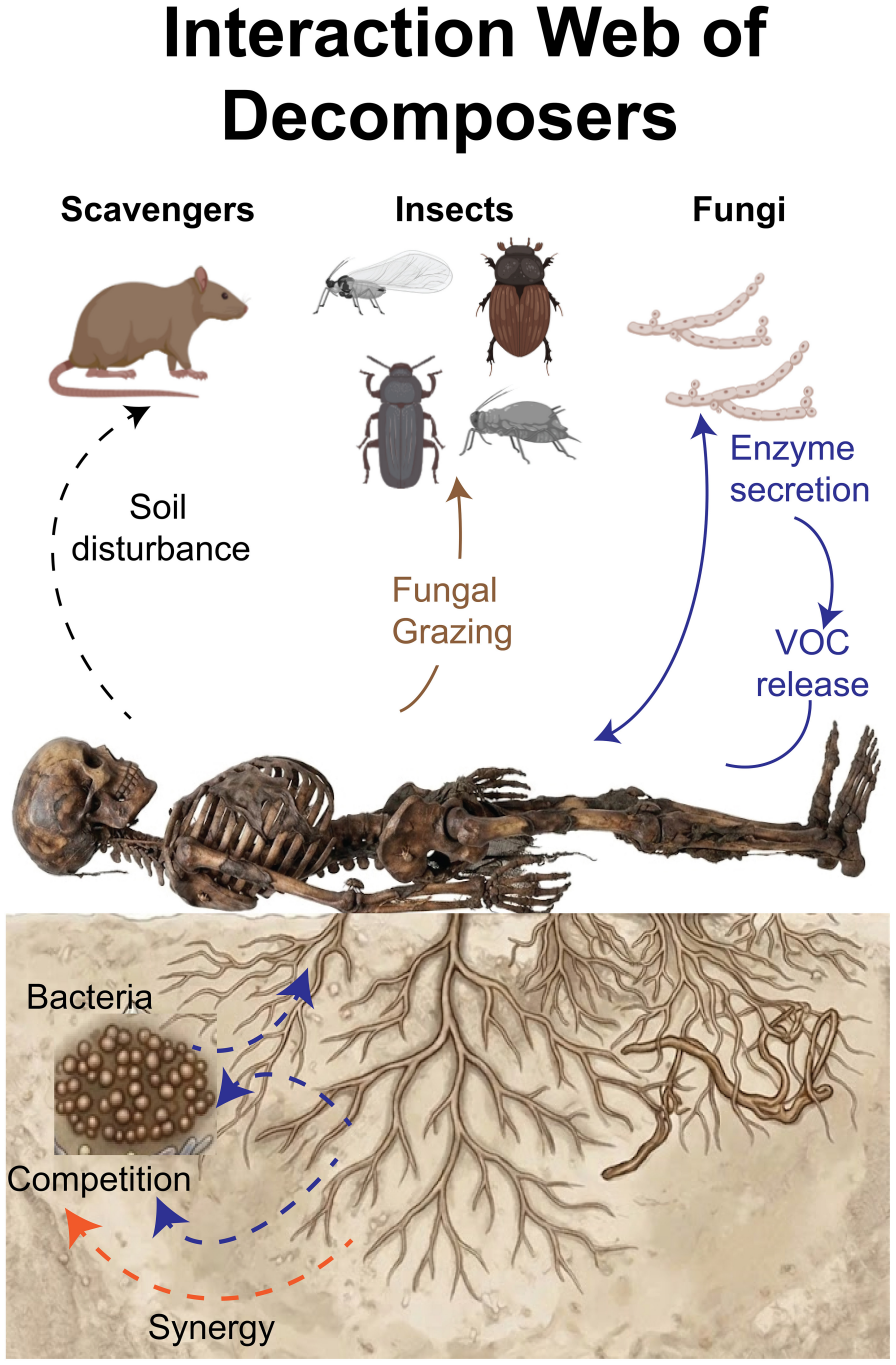
Ecological interaction web surrounding a decomposing cadaver, illustrating dynamic relationships among fungi (enzyme secretion, VOC emission), bacteria (competitive and synergistic interactions), insects (spore dispersal and fungal grazing), and scavengers (soil disturbance). Illustrative conceptual diagram created with AI assistance; see text for supporting citations and evidence. This figure is intended to generate testable hypotheses, not to present quantitative empirical data.

Mushrooms establish complex ecological relationships with bacteria, insects, and scavengers during cadaver decomposition, forming a dynamic network that directly impacts forensic evidence. These interactions can accelerate, delay, or alter decomposition patterns in predictable ways, offering new avenues for forensic analysis (Figure 6).

#### 2.3.1 Fungal-Bacterial interactions

Basidiomycota have intricated, interactive relationships with bacteria in the decay of cadavers, through competitive and cooperative processes that profoundly shape the process of decay. Similar interdisciplinary associations are found in forensic botany, where plant-fungal-microbial associations, such as bryophytes and fungi in soils, serve as trace evidence in forensic contexts (Raje et al., 2022). These complex interactions underscore the need to move beyond single-kingdom forensic models. The establishment of integrated multi-kingdom decomposition models (MKDMs) that quantitatively account for fungal-bacterial-arthropod feedback loops is the logical and necessary next step for the field.

A few cadaver-related mushrooms, for example, the *Coprinopsis* and *Gymnopus* genera, also harbor active antimicrobial compounds, such as coprinol, that form localized zones of fungal dominance by suppressing competing microorganisms (Stamets, 2005). This is in agreement with Vidona et al. (2025), who documented competition among microbes for substrate in the cadavers where fungi dominate bacteria for the same substrate in nitrogen-rich environments (Carter and Tibbett, 2008; Haelewaters, 2013). This is followed by nutrient competition processes: nitrogen-fixing microorganisms, such as *Pseudomonas*, are the prevalent initial decomposers, but are later succeeded by fungi such as *Hebeloma* as proteins degrade into more bioavailable amino acids (Pechal et al., 2018).

These fungal-bacterial interactions during decomposition have equivalents in microbial succession patterns observed in latent fingerprints. Recent 16S rRNA sequencing studies of fingerprint microbiota show that, while dominant bacterial phyla remain rather stable for about 7 days after deposition, minor phyla such as Deinococcus-Thermus exhibit predicted variation by 14 to 21 days (De Alcaraz-Fossoul et al., 2023). Just as *Hebeloma's* nitrogen-induced ecology in gravesoils, these fingerprint-associated microbes exhibit stage-specific succession. This suggests a broader forensic idea: microbial populations—in decaying remains or trace evidence may function as biological clocks, especially when conventional markers like insects or culturable bacteria are absent or degraded.

Remarkably, synergistic and competitive interactions co-occur. *Clitocybe* species, for example, were observed to synergize with proteolytic bacteria in breaking down collagen via a sequential process in which bacteria degrade tissues into small peptides, which are then metabolized by fungi (Boddy and Jones, 2008). Chemical mediation is not confined to immediate microbial interactions; *Hebeloma* spp., as ammonia fungi, alter their microenvironment by alkalinizing it through ammonia metabolism. Such a mechanism suppresses acidophilous bacteria while attracting nitrophilous insects to the gravesoil (Tibbett and Carter, 2003). Field observations by Haelewaters (2013) also indicate the forensic significance of *Hebeloma* species, such as *H. vinosophyllum* and *H. syrjense*, in that fruiting is anticipated near concealed corpses, earning them the nickname “corpse finders.” While such a species always indicates nitrogen enrichment from decomposition, its specific use in PMI estimation or grave localization remains to be explored. Several critical parameters, including soil composition (riparian vs. sandy soils), climatic variations (humidity and temperature), and carcass mass, greatly influence patterns of fungal colonization. For instance, *Hebeloma* sporocarps will appear earlier under high-nitrogen conditions but are suppressed under anaerobic burial conditions. Conducting controlled experiments while accounting for these factors, combined with more sophisticated metabarcoding methods like those used by Giampaoli et al. (2021), can advance *Hebeloma* from an ecological curiosity to a workable forensic biomarker admissible in court.

In addition, postputrefaction fungi, such as *Coprinopsis* species, produce volatile organic compounds (VOCs), including geosmin, which act as chemical signals to recruit necrophagous beetles (Pacioni et al., 2015). These various interactions give rise to complex feedback involving fungal activity, soil chemistry, and arthropod colonization patterns. Present metatranscriptomic evidence indicates that the foregoing feedbacks are also regulated by inherent characteristics such as the cadaver's body mass index (BMI). High-BMI material would see increased nitrogen flux, promoting greater colonization by ammonia fungi such as *Hebeloma* spp. compared to low-BMI remains. These BMI-dependent alterations in fungal-bacterial-arthropod networks illustrate the need to incorporate mass-adjusted decomposition models into forensic science (Mason, 2022).

#### 2.3.2 Fungal-Insect interactions

Insects are important vectors for fungal spores, and at the same time, they are impacted by fungal action in several ways. For example, Diptera larvae such as Lucilia blowflies feed on the mycelium of fungi such as *Mycena* and *Psilocybe*, thereby unintentionally spreading spores through their intestines (Sagara, 1995). Similarly, dermestid beetles tend to browse tissues infested with *Coprinopsis* and leave distinctive feeding patterns that can be followed. Fungal species also use chemical signaling to control insect behavior: *Pholiota* and *Entoloma*, among other genera, release VOCs such as geosmin, which recruit necrophagous insects to rotting corpses (Pacioni et al., 2015). *Gymnopus* releases sesquiterpenes that deter particular beetle taxa, impacting the timing and order of insect colonization of cadavers.

Apart from chemical cues, insects also modify the physical environment in ways that are conducive to fungi. For instance, tunneling activities of beetles aerate cadaver tissues and raise oxygen levels in areas that would otherwise remain low. Aerating increases mushroom growth, particularly during late stages of decay (Hawksworth and Wiltshire, 2015). Although fruiting of older Basidiomycota, such as ectomycorrhizal fungi, typically occurs several years after decomposition, they can still indirectly benefit from insect-mediated aeration of soil and tissues (Tranchida et al., 2021).

In extant arthropods, nematode populations also undergo continued changes in decomposition islands lasting longer than 4 years since deposition. This multi-year timescale aligns with the multi-year fruiting periods of ammonia fungi such as *Hebeloma* spp. and demonstrates close trophic interconnections between nematodes and fungi. Mycophagous nematodes, including members of the family Rhabditidae, follow closely behind the fungus succession, while microbivorous nematodes respond to the nitrogen fluxes that cause fungal sporocarp growth. Seasonal high-resolution observations have documented synchronous maxima in nematode densities, namely bacterivorous Diplogasteridae, coinciding with sporocarp emergence of the fungi. The two groups are strongly correlated (p < 0.01) with thermal sum, measured as accumulated degree days (ADD), and ammonium peaks in gravesoils, indicating a closely associated ecological interaction between nematode populations and fungal growth during decay (Taylor, 2020).

### 2.4 Habitat-specific constraints on basidiomycota detection

The utility of Basidiomycota as forensic biomarkers depends heavily on habitat. Global surveys of soil fungal communities indicate that Ascomycota, rather than Basidiomycota, dominate most terrestrial ecosystems. Basidiomycota exhibit significant environmental limitations, including reduced biomass under flooding and restriction to specific natural areas (Tedersoo et al., 2014). In contrast, aquatic systems present a markedly different scenario. Kaszubinski et al. (2022) found that submerged bones exhibit minimal Basidiomycota succession, with bacterial communities providing a more reliable temporal resolution for postmortem submersion intervals. Additionally, marine and freshwater environments contain distinct, habitat-specific fungal assemblages dominated by Ascomycota and Chytridiomycota, rather than the nitrogen-responsive Basidiomycota prevalent in terrestrial decomposition (Grossart et al., 2019). Consequently, the forensic applicability of Basidiomycota biomarkers is greatest in terrestrial surface decomposition, particularly in temperate forest soils where ammonia fungi are well documented. Application to aquatic environments or flooded terrestrial systems requires independent investigation and should not be inferred from terrestrial patterns.

## 3 Scavenger-mediated dispersal

Vertebrate scavengers play a critical role in the spread of forensic fungi. For instance, ammonia fungi spores, such as *Hebeloma*, stick to mammalian scavenger fur and bodies, including those of rodents and foxes, which unintentionally disperse these spores to new sites of decomposition, facilitating the colonization of the landscape by fungi (Fu et al., 2019). In addition, scavenging behavior, such as soil disturbance and digging, unearths concealed mycelial networks that had been buried in the ground. Mechanical stress induces mushroom fruiting in fungal species such as *Mycena* and *Clitocybe* at disturbed burial sites; hence, these fungi are readily visible and available in the environment (Sagara et al., 2008).

These types of fungal dispersal mechanisms involving scavengers have several direct forensic applications. Insect larvae feeding on *Coprinopsis* species, for example, indicate active stages of decay, while communities dominated by *Gymnopus* mushrooms indicate subsequent stages of decomposition. Moreover, aggregations of mushroom-stimulated necrophagous insects such as beetles aggregating over volatile substances emitted by *Pholiota* can act as biological indicators for burial site occurrence. Along with ecologic information, the antimicrobial compounds produced by mushrooms, including coprinol, can influence preservation or alteration of toxicological samples within decayed tissues and thus offer a biochemical aspect to forensic science.

A deep understanding of these intricate fungal-insect-scavenger relationships enables forensic scientists to situate mushroom evidence within the broader ecological context of decomposition. This approach moves beyond dichotomous observations to harmonious, integrated forensic models that consider multi-kingdom relationships. To further advance this field, measuring these associations in controlled experimental conditions will set protocol standards for crime scene evaluation, improve postmortem interval estimates, and enhance grave detection protocols.

## 4 Soil mycobiomes as trace evidence in homicide investigations

Fungal communities that come with decomposition offer unique advantages as trace evidence in forensic science, particularly due to their remarkable resilience in soil environments. While bacterial traces easily degrade, the mycelium networks of ammonia fungi such as *Hebeloma* may remain intact for years in CDIs and yield enduring biological signatures of burial locations (Sagara, 1995). This long-term stability has been supplemented by developments in molecular technology over the last few years, as a controlled trial undertaken in Istanbul demonstrated that Illumina NovaSeq ITS1 sequencing of fungal communities achieved 83% success in detecting corresponding soil samples in criminal case crime scenes compared to bacterial microbiome analyses (Karadayi, 2021). Figure 6 presents a step-by-step operational framework outlining the procedures for gathering and analyzing fungal evidence in forensic cases.

The forensic application of soil mycobiomes works through two complementary but distinct mechanisms. First, the spatial coordination capability is demonstrated by CDI-specific fungal taxa, which include both long-lasting mycelium and sticky spores. *Hebeloma* mycelium creates areas of concentrated nitrogen in burial soils, while *Coprinopsis* species' spores firmly bind to clothing and footwear, providing transfer evidence traceable to suspects to crime scenes. Second, temporal resolution is obtained from successional patterns in fungal communities, allowing scientists to estimate the postmortem interval by identifying the growth stages of certain fungi. Ecological context is crucial for understanding fungal trace evidence. Terrestrial environments have high potential for long-term fungal signatures, whereas aquatic environments pose other challenges. A study by Kaszubinski et al. (2022) showed that submerged bones exhibit low Basidiomycota succession, along with early colonization stages, with bacterial communities providing higher temporal resolution for postmortem submersion durations.

This habitat-based heterogeneity underscores the need for ecosystem-specific approaches in forensic mycology. Finley et al.'s (2015) pioneering work on bacterial microbial clocks in gravesoils provides a model for the derivation of similar applications with fungal populations. Their findings on the impact of soil texture and moisture levels on microbial activity directly address our understanding of the environmental factors controlling forensic-relevant fungi like *H. radicosum*. This ecological strategy highlights the necessity of standardized protocols that include parallel sequencing of entire mycobiomes using ITS markers and Basidiomycota-specific gene targets, as well as the traditional morphological determination of sporocarps from soil samples.

The potential of soil mycobiomes is illustrated by a case from Buenos Aires (Tranchida and Cabello, 2017), where *Talaromyces*-dominated gravesoils were reportedly matched to a suspect's shovel. While promising, such case reports highlight the need for more extensive validation studies and standardized protocols to ensure courtroom admissibility. To best utilize these advances in forensic practice, the creation of large regional databases recording CDI fungal signatures will be required, augmented by the addition of mycological examination to standard forensic procedure. This technique applies Locard's Exchange Principle to the analysis of fungal evidence, providing new means to link suspects to crime scenes even when no degraded or missing conventional evidence is present.

## 5 Challenges in implementing fungal evidence in forensic investigations

**Figure 7:**
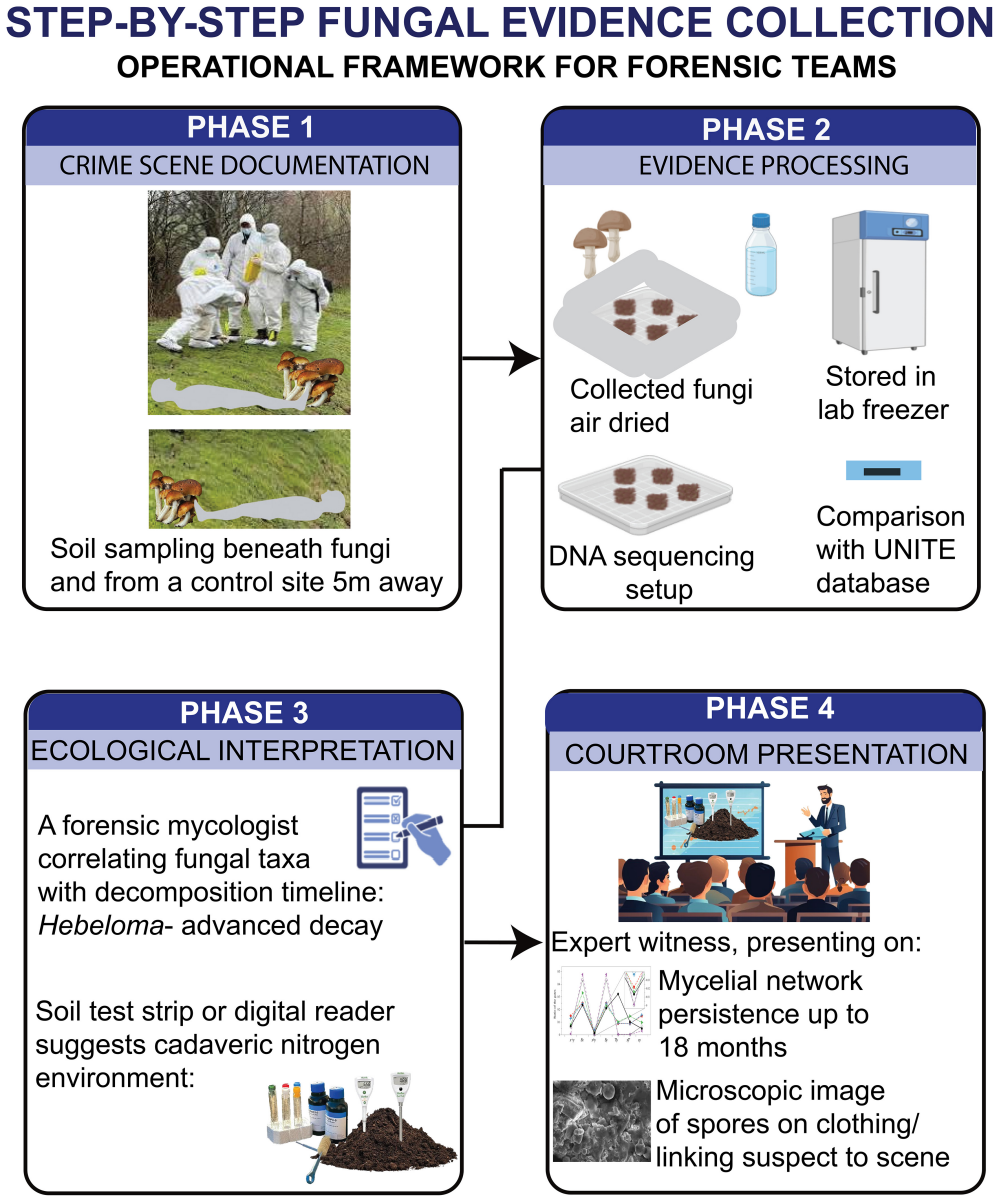
Step-by-step operational framework for fungal evidence collection and analysis in forensic investigations. Illustrative conceptual diagram created with AI assistance; see text for supporting citations and evidence. This figure is intended to generate testable hypotheses, not to present quantitative empirical data.

Whereas previous reviews of forensic mycology have tended to describe microbial communities (Hyde et al., 2017) or undefined decomposition processes, the current work establishes macroscopic Basidiomycota as the next frontier for forensic science on three distinct advantages unavailable in existing methodologies. First, their surface sporocarps enable field identification without the need for laboratory cultivation. Second, their mycelia networks and chemical signatures persist longer than insect cadavers or volatile organic compounds. Third, their enzyme-specific breakdown patterns provide biochemical decay timelines that complement existing entomological and microbiological methods. These unique characteristics position Basidiomycota as transformative biomarkers, but there are several implementation challenges to address (Figure 7).

Taxonomic acceptance of forensically significant fungi is an impediment to utilization. Many of the Basidiomycota species that are of forensic significance, including key ammonia fungi such as *H. vinosophyllum*, have subtle morphological variations that require experienced recognition - a resource not likely to be available in most forensic science units. Although molecular methods are essential, current internal transcribed spacer (ITS) reference databases contain very few sequences for decomposition fungi, particularly for key ammonia fungi (Schoch et al., 2012; Nilsson et al., 2019). Furthermore, no forensic-validated pipeline currently includes reproducibility testing or satisfies Daubert standards for admissibility. Studies of fungal identification report error rates ranging from 0 to 70 percent, depending on marker selection and database completeness (Blaalid et al., 2013), with misidentification rates reaching up to 16.6 percent in certain contexts. The morphological key presented in Figure 5 serves as a complementary field tool; however, integration with molecular methods and quantification of error rates remain underdeveloped.

Legal admissibility is also a major stumbling block, although there is precedent for the promise of fungal evidence. *H. radicosum* sporocarps were successfully employed to assist in PMI estimation in a Swiss homicide case, and Japanese researchers have utilized *Coprinopsis* species to locate concealed graves. Broader judicial acceptance, however, will be contingent upon standardized protocols meeting Daubert reliability standards. To meet legal admissibility standards, the discipline must develop: (1) standardized evidence recovery protocols that dictate the photographic, spatial, and physical collection of sporocarps to preserve delicate morphological features; (2) validated molecular assays with empirically defined error rates and limits of detection for key biomarker species; and (3) statistically robust successional models that incorporate microenvironmental covariates and provide confidence intervals for PMI estimates. These require unprecedented interdisciplinary cooperation. Mycologists must work in tandem with forensic experts to translate theoretical ecological insights into field-relevant protocols, while legal experts help determine the standards of evidence. This parallels the path toward acceptance of forensic entomology, though Basidiomycota offer unique advantages: enzymatic profiles (e.g., keratinolysis) provide biochemical timelines unlike insect development markers, and nitrogen-based fruiting bodies leave traceable crime scene markers when everything else degrades. With these challenges addressed, Basidiomycota forensics has the potential to revolutionize death investigation, particularly in cold cases or extreme environments where traditional methods fail. Their dual function as both ecological clocks and space markers is a novel synthesis that could make fungal evidence crucial to 21^st^-century forensic science.

### 5.1 Methodological limitations of this synthesis

Several methodological constraints temper the strength of our conclusions and should guide the interpretation of this review.

#### 5.1.1 Absence of systematic review methodology

This synthesis did not employ pre-registered search criteria, formal inclusion and exclusion protocols, or bias assessment, all of which are now considered essential for minimizing selection bias in ecological syntheses (Gurevitch et al., 2018). Consequently, the possibility of selection bias toward studies supporting the forensic utility of Basidiomycota cannot be excluded. This concern is well documented in ecological research, where 38-50% of meta-analyses demonstrate significant publication bias (Jennions and Møller, 2002).

#### 5.1.2 Lack of quantitative meta-analysis

The forensic fungal literature currently lacks standardized reporting of successional timelines, environmental covariates, and detection probabilities, all of which are necessary for robust meta-analysis with heterogeneity quantification. In the absence of effect-size calculations across studies, claims of “predictability” and “persistence” remain qualitative observations rather than quantitatively validated metrics. This gap should be addressed by future research through coordinated, multi-site studies employing harmonized methodologies.

#### 5.1.3 Conceptual figure status

All figures in this review are schematic integrations of published observations and ecological theory. Their purpose is to generate testable hypotheses rather than to present quantitative empirical data. Each figure caption explicitly states this conceptual intent (see Figures 1–7 captions).

#### 5.1.4 Need for formal evidence synthesis

Future bias-assessed meta-analyses with quantified heterogeneity are necessary to validate claims of predictability and persistence across habitats. Until such standardized syntheses are available, the conclusions of this review remain provisional and hypothesis-generating rather than definitive.

#### 5.1.5 Conceptual status of spatial models

The spatial relationships depicted in Figure 2, including vertical stratification and nutrient diffusion gradients, remain conceptual. No published study has integrated ITS-based fungal community profiling with measured ammonium or pH gradients in vertical CDI transects. This gap highlights the need for empirical validation of conceptual models in forensic applications.

#### 5.1.6 Lack of functional validation in CDI contexts

The biochemical pathways depicted in Figure 4 are conceptually derived from documented fungal enzyme repertoires established in non-forensic environments. However, no studies to date have provided CDI-specific genomes, transcripts, proteins, or metabolites directly linked to Basidiomycota activity during decomposition. The only cadaveric fungal study cited (Fu et al., 2019) showed a decline in Basidiomycota during decomposition, contradicting the assumption of functional dominance. In the absence of taxon-resolved functional meta-omics from actual CDI soils, the mechanistic attributions in Figure 4 remain speculative, and claims that these pathways ‘elucidate timelines' are not currently supported by evidence. This gap underscores the need for targeted metatranscriptomic and metabolomic investigations.

#### 5.1.7 Lack of persistence benchmarks

A significant gap in the forensic application of Basidiomycota is the absence of quantitative persistence data. No longitudinal studies have provided survival curves or detection-window comparisons for sporocarps relative to insect evidence or bacterial signatures. Assertions that fungal evidence ‘outlasts' insect and bacterial evidence remain qualitative and anecdotal. Systematic, cross-biome studies are required to establish empirical benchmarks for sporocarp persistence under varying environmental conditions, thereby informing evidence triage decisions at crime scenes.

## 6 Discussion and future directions

This review synthesizes current evidence regarding the forensic application of macroscopic Basidiomycota, highlighting both considerable potential and notable knowledge gaps. Key findings include the observation that ammonia fungi, such as *Hebeloma* and *Coprinopsis*, display predictable nitrogen-responsive fruiting patterns during decomposition, which may serve as biomarkers for grave localization and PMI estimation. Although enzymatic capabilities for degrading recalcitrant tissues such as keratin and collagen are well documented in non-forensic contexts, these functions lack validation in CDI settings. Habitat-specific constraints limit the universal applicability of these fungi, with terrestrial surface decomposition identified as the most promising context. Significant methodological challenges, such as the lack of standardized protocols, incomplete reference databases, and insufficient quantitative validation, currently prevent admissibility in court. Building on this synthesis, we propose future directions to advance Basidiomycota from promising ecological indicators to legally admissible forensic biomarkers.

As forensic mycology matures from a developing discipline to a mature forensic science, this review anticipates three paradigm-shifting frameworks that will render Basidiomycota principal tools for death investigations. These connected initiatives transcend current setbacks while creating new standards for evidence validity and courtroom admissibility. A primary future direction is the development of interpretable machine learning models for PMI estimation. In response to groundbreaking work by (Mason et al. 2024), these computer packages will contrast the elaborate phenologies of sporocarps, particularly the fruiting curves of ammonia fungi such as *Hebeloma* species, with successional mycobiome dynamics in soils and important environmental covariates, such as temperature gradients, soil pH variations, and precipitation records. Early implementations have been very promising, with proof-of-concept neural networks achieving 89% prediction accuracy for decomposition phases from multi-year fungal succession of controlled grave soil experiments. The technique is an advancement over traditional linear models by virtue of its ability to model the nonlinear dynamics of fungal colonization patterns and post-mortem time.

To overcome longstanding challenges in taxonomic identification, we propose establishing the Forensic Fungi ITS Database (FFD) as a global, gold-standard reference. It is an international consortium-based initiative that will methodically curate ITS sequences from voucher specimens directly linked with decomposition research, as well as geo-referenced occurrence data for primary biomarker species such as *Coprinopsis atramentaria*. Critically, this database must be accompanied by validated identification pipelines that include: reproducibility testing across laboratories, empirically defined error rates and limits of detection for key biomarker species, blind validation studies to establish sensitivity and specificity, and proficiency testing programs for forensic practitioners. These elements are essential for meeting Daubert admissibility standards and ensuring that fungal evidence withstands legal scrutiny. The database will contain morphological keys designed specifically for field technicians and will integrate easily with mobile sequencing platforms, enabling instant, correct species identification at the crime scene. By creating this specialist tool, the forensic community can avoid the current reliance on ecological databases lacking decomposition-specific metadata, thereby bypassing one of the primary barriers to the courtroom admissibility of fungal evidence.

Potentially the most ambitious development is to build Multi-Kingdom Decomposition Models (MKDM) that integrate fungal succession chronologies with established insect life-cycle information and bacterial OTU wave patterns. These combined models will both identify and quantify important cross-kingdom interaction nodes, such as the *Pseudogymnoascus* fungi's suppression of blowfly larval development, and incorporate habitat-specific weighting factors that adjust the evidentiary value of fungal data relative to environmental conditions. The statistical model design will adjust automatically the relative credibility of fungal evidence to other biological markers with respect to stage of decomposition, climatic zone, and ecosystem type. A hypothetical application in a cold case scenario demonstrates the framework's promise: combining *Hebeloma* sporocarp distribution (to refine PMI), ITS sequencing of soil from a suspect's footwear (for spatial linking), and a multi-kingdom model that weights fungal evidence more heavily in arid conditions could powerfully reconstruct a crime.

The application of these frameworks will have synergistic effects across forensic practice. The FFD provides the taxonomic foundation required for courtroom-acceptable evidence, and machine learning models give quantifiable confidence bounds to fungal-based time since death estimates. Meanwhile, the MKDM system provides robust evidence triangulation across biological systems, thereby circumventing the disadvantages of single-method approaches. Collectively, they position Basidiomycota not only as adjunct biomarkers but also as lead forensic markers capable of yielding unique insights unobtainable with standard entomological or microbiological methods.

As these new methods mature via consortial research and field testing, the coming decade will witness a paradigm shift in forensic science. The addition of fungal evidence through these quantitative, standardized methods has the potential to take “mycological time since death estimation” out of the realm of experimentation into routine forensic practice, particularly for cold cases and extreme environments where older methods fail. This breakthrough will require sustained investment in interdisciplinary research and infrastructure development, but the potential rewards - enhanced investigations, more powerful courtroom evidence, and justice for more victims - make it one of the most exciting frontiers in modern forensic science.

### 6.1 Formal evidence synthesis and meta-analysis

A critical priority identified in this review is the implementation of formal evidence synthesis within forensic mycology. Existing literature is dominated by observational case studies and conceptual frameworks, lacking the quantitative synthesis required for courtroom admissibility.

Future research should address the following objectives:

(1) Establish standardized reporting protocols for fungal succession studies, mandating documentation of environmental covariates (temperature, precipitation, soil pH, moisture), detection methods, and successional timelines to facilitate cross-study comparability. In the absence of standardized data reporting, meta-analysis cannot be conducted.
(2) Conduct meta-analyses that assess bias and quantify heterogeneity to validate claims regarding predictability and persistence across habitats. These analyses should formally test for publication bias using methods such as funnel plots and trim-and-fill analysis (Jennions and Møller, 2002), and apply random-effects models to account for variation between studies (Gurevitch et al., 2018).
(3) Develop habitat-stratified reference databases documenting effect sizes for fungal-indicator performance across terrestrial, aquatic, arid, and cold environments. These databases should include confidence intervals and measures of heterogeneity to establish statistically robust baselines for forensic interpretation.
(4) Design controlled mesocosm experiments with urea-amended soils to determine quantitative ammonium thresholds for fruiting initiation in key forensic species (such as *H. vinosophyllum* and *Coprinopsis* species) under varying temperature and moisture regimes. These studies should systematically manipulate pH, soil texture, and humidity to develop predictive models with quantified sensitivity and specificity for forensic applications.
(5) Perform power analyses to determine the minimum sample sizes necessary for detecting fungal succession patterns with sufficient statistical certainty across diverse environmental contexts.

In the absence of formal evidence syntheses, claims regarding Basidiomycota as forensic biomarkers should be regarded as provisional and hypothesis-generating rather than definitive. The forensic mycology community should prioritize quantitative methodologies to facilitate the transition from ecological observation to courtroom-admissible science.

### 6.2 Functional meta-omics in CDI contexts

A key priority identified in this review is validating biochemical pathways in real-world CDI environments. Future investigations should utilize integrated meta-omics methodologies to:

(1) Characterize CDI-specific metagenomes to determine which fungal taxa harbor genes encoding enzymes relevant to decomposition, such as amine oxidases, keratinases, and collagenases.
(2) Conduct metatranscriptomic analyses to identify genes that are actively expressed at various decomposition stages, thereby demonstrating functional activity rather than simple gene presence.
(3) Perform metabolomic profiling to quantify volatile organic compounds, such as geosmin, and associate their production with particular fungal taxa and decomposition phases.
(4) Integrate proteomic and metabolomic data with ITS-based community profiling to facilitate taxon-resolved functional inference across vertical strata within CDIs.

These studies have the potential to convert the conceptual pathways illustrated in Figure 4 from hypothetical mechanisms into empirically validated forensic tools with established temporal and spatial dynamics.

## 7 Conclusion

The study of Basidiomycota as forensic biomarkers represents a promising frontier for expanding the death investigation arsenal, contingent upon rigorous validation through standardized methodologies and formal evidence synthesis. Basidiomycota have much to offer, from routine colonization of cadavers to the ability to survive where other biological evidence degrades. However, with strong evidence of their potential all around, fungal forensics is an untapped area of research suspended in limbo between ecological potential and everyday applicability. The road forward will involve action from various stakeholders. We call upon forensic agencies to acknowledge the evidentiary quality of mycological evidence by incorporating fungal identification training into forensic education programs and establishing best-practice standards for evidence collection. Standardization is imperative without established protocols for recording sporocarps, storing samples, and processing fungal succession patterns; this evidence will never be admissible in court.

Its priority, for the scientific community, must be to bridge knowledge gaps through synergistic efforts. There is a pressing requirement for long-term field experiments crossing multiple biomes to refine models of fungal succession across a range of climatic and edaphic conditions. These experiments need to adopt standardized procedures to facilitate cross-site comparisons and to cover the entire gradient of decomposition regimes, from surface detritus to buried corpses, across arid to wetland habitats. The development of infrastructure to facilitate forensic use is also required. A highly advanced, freely accessible database of forensically important fungi would serve as the taxonomic platform for secure identification, and machine learning algorithms could translate ecological patterns into useful investigative leads. Such tools would need to be developed in collaboration among mycologists, forensic scientists, and data scientists, with close attention to meeting both biological and legal needs. The reward is well worth the effort. In an era when criminals are going out of their way to use remote locations and long post-mortem intervals to avoid detection, fungal biomarkers are a powerful tool for foiling such plans. The ability of fungal biomarkers to detect indicators of decay in low-nutrient soil, cold temperatures, or water environments that are challenging for traditional methods makes them particularly well-suited for cold cases and mass disaster situations.

As we stand at the threshold of this new discipline, the choice is ours: do we relegate fungi to the forensic science sidebar, or welcome them to join us as honored partners in our pursuit of justice? The future is ours, in our will to support the research, training, and infrastructure that will enable us to unleash their full potential. In doing so, we not only live up to the promise of scientific discovery but also to our fundamental obligation to become the voice of the voiceless, who are no longer capable of speaking up for themselves. Let this be our challenge, forensic scientists, practitioners, and funding agencies, to bring fungal forensics from promise to practice. The silent witness waits; realizing its evidentiary potential requires sustained investment in standardized research, validation studies, and formal evidence syntheses that will translate ecological observation into courtroom-admissible science.

## References

[B1] Amendt J. Krettek R. Zehner R. (2004). Forensic entomology. Naturwissenschaften 91, 51–65. doi: 10.1007/s00114-003-0493-5 14991142

[B2] Anderson I. C. Genney D. R. Alexander I. J. (2013). Fine-scale diversity and distribution of ectomycorrhizal fungal mycelium in a Scots pine forest. New Phytol. 201, 1423–1430. doi: 10.1111/nph.12637 24345261

[B3] Baldrian P. (2006). Fungal laccases - occurrence and properties. FEMS Microbiol. Rev. 30, 215–242. doi: 10.1111/j.1574-4976.2005.00010.x 16472305

[B4] Blaalid R. Kumar S. Nilsson R. H. Abarenkov K. Kirk P. M. Kauserud H. . (2013). ITS1 versus ITS2 as DNA metabarcodes for fungi. Mol. Ecol. Resour. 13, 218–224. doi: 10.1111/1755-0998.12065 23350562

[B5] Boddy L. Jones T. H. (2008). “Interactions between basidiomycota and invertebrates,” in British Mycological Society Symposia Series, Vol. 28 (London: Academic Press), 155–179. doi: 10.1016/S0275-0287(08)80011-2

[B6] Callahan B. J. McMurdie P. J. Holmes S. P. (2017). Exact sequence variants should replace operational taxonomic units in marker-gene data analysis. ISME J. 11, 2639–2643. doi: 10.1038/ismej.2017.119 28731476 PMC5702726

[B7] Carter D. O. Tibbett M. (2008). “Cadaver decomposition and soil: processes,” in Soil Analysis in Forensic Taphonomy: Chemical and Biological Effects of Buried Human Remains (Boca Raton, FL: CRC Press), 29–51. doi: 10.1201/9781420069921.ch2

[B8] Carter D. O. Yellowlees D. Tibbett M. (2007). Cadaver decomposition in terrestrial ecosystems. Naturwissenschaften 94, 12–24. doi: 10.1007/s00114-006-0159-1 17091303

[B9] Chimutsa M. Olakanye A. O. Thompson T. J. Ralebitso-Senior T. K. (2015). Soil fungal community shift evaluation as a potential cadaver decomposition indicator. Forensic Sci. Int. 257, 155–159. doi: 10.1016/j.forsciint.08.005 26322496

[B10] De Alcaraz-Fossoul J. Wang Y. Liu R. Mancenido M. Marshall P.A. Núñez C. . (2023). Microbes in fingerprints: a source for dating crime evidence?. Forensic Sci. Int. Genet. 65:102883. doi: 10.1016/j.fsigen.2023.102883 37120981

[B11] Deel H. Emmons A. L. Kiely J. Damann F. E. Carter D. O. Lynne A. . (2021). A pilot study of microbial succession in human rib skeletal remains during terrestrial decomposition. MSphere 6:e00455. doi: 10.1128/mSphere.00455-21 34259562 PMC8386422

[B12] Emmons A. L. Mundorff A. Z. Keenan S. W. Davoren J. Andronowski J. Carter D. O. . (2020). Characterizing the postmortem human bone microbiome from surface-decomposed remains. PLoS ONE 15:e0218636. doi: 10.1371/journal.pone.0218636 32639969 PMC7343130

[B13] Finley S. J. Benbow M. E. Javan G. T. (2015). Potential applications of soil microbial ecology and next-generation sequencing in criminal investigations. Appl. Soil Ecol. 88, 69–78. doi: 10.1016/j.apsoil.01.001

[B14] Floudas D. Binder M. Riley R. Barry K. Blanchette R. A. Henrissat B. . (2012). The Paleozoic origin of enzymatic lignin decomposition reconstructed from 31 fungal genomes. Science 336, 1715–1719. doi: 10.1126/science.1221748 22745431

[B15] Fu X. Guo J. Finkelbergs D. He J. Zha L. Guo Y. . (2019). Fungal succession during mammalian cadaver decomposition and potential forensic implications. Sci. Rep. 9:12907. doi: 10.1038/s41598-019-49361-0 31501472 PMC6733900

[B16] Fukiharu T. Hongo T. (1995). Ammonia fungi of Iriomote island in the southern Ryukyus, Japan and a new ammonia fungus, Hebeloma luchuense. Mycoscience 36, 425–430. doi: 10.1007/BF02268627

[B17] Fukiharu T. Osaku K. Iguchi K. Masahiko A. (2000a). Occurrence of ammonia fungi on the forest ground after decomposition of a dog carcass. Nat. Hist. Res. 6, 9–14.

[B18] Fukiharu T. Yokoyama G. Oba T. (2000b). Occurrence of *Hebeloma vinosophyllum* on the forest ground after decomposition of crow carcass. Mycoscience 41, 401–402. doi: 10.1007/BF02463954

[B19] Giampaoli S. De Vittori E. Barni F. Anselmo A. Rinaldi T. Baldi M. . (2021). DNA metabarcoding of forensic mycological samples. Egypt. J. For. Sci. 11:7. doi: 10.1186/s41935-021-00221-x

[B20] Grossart H. P. Van den Wyngaert S. Kagami M. Wurzbacher C. Cunliffe M. Rojas-Jimenez K. . (2019). Fungi in aquatic ecosystems. Nat. Rev. Microbiol. 17, 339–354. doi: 10.1038/s41579-019-0175-8 30872817

[B21] Gurevitch J. Koricheva J. Nakagawa S. Stewart G. (2018). Meta-analysis and the science of research synthesis. Nature 555, 175–182. doi: 10.1038/nature25753 29517004

[B22] Haelewaters D. (2013). *Hebeloma*, pioneer genus in forensic mycology. Fungi 6, 47–48.

[B23] Hawksworth D. L. Wiltshire P. E. (2011). Forensic mycology: the use of fungi in criminal investigations. Forensic Sci. Int. 206:1. doi: 10.1016/j.forsciint.06.012 20634009

[B24] Hawksworth D. L. Wiltshire P. E. (2015). Forensic mycology: current perspectives. Res. Rep. For. Med. Sci. 5, 75–83. doi: 10.2147/RRFMS.S83169

[B25] Hibbett D. S. Donoghue M. J. (2001). Analysis of character correlations among wood decay mechanisms, mating systems, and substrate ranges in homobasidiomycetes. Syst. Biol. 50, 215–242. doi: 10.1080/10635150151125879 12116929

[B26] Hilton R. N. (1978). The ghoul fungus, Hebeloma sp. ined. Trans. Mycol. Soc. Japan. 19:418.

[B27] Hitosugi M. Ishii K. Yaguchi T. Chigusa Y. Kurosu A. Kido M. . (2006). Case report: fungi can be a useful forensic tool. Legal Med. 8, 240–242. doi: 10.1016/j.legalmed.04.005 16798051

[B28] Hyde E. R. Metcalf J. L. Bucheli S. R. Lynne A. M. Knight R. (2017). Microbial communities associated with decomposing corpses. Forensic Microbiol. 245–273. doi: 10.1002/9781119062585.ch10

[B29] Inácio F. D. Martins A. F. Contato A. G. Brugnari T. Peralta R. M. de Souza C. G. . (2017). (2018). Biodegradation of human keratin by protease from the basidiomycete *Pleurotus pulmonarius*. Int. Biodeterior. 127, 124–129. doi: 10.1016/j.ibiod.11.010

[B30] Ishii K. Hitosugi M. Kido M. Yaguchi T. Nishimura K. Hosoya T. . (2006). An analysis of fungi detected in human cadavers. Leg. Med. 8, 188–190. doi: 10.1016/j.legalmed.12.006 16516528

[B31] Jasim N. O. (2021). Forensic mycology: fungal evidences in forensic analysis - a review. Int. J. Pharm. Qual. Assur. 12, 100–103. doi: 10.25258/ijpqa.12.2.15

[B32] Jennions M. D. Møller A. P. (2002). Publication bias in ecology and evolution: an empirical assessment using the “trim and fill” method. Biol. Rev. 77, 211–222. doi: 10.1017/S1464793101005875 12056747

[B33] Karadayi S. (2021). Assessment of the link between evidence and crime scene through soil bacterial and fungal microbiome: a mock case in forensic study. Forensic Sci. Int. 329:111060. doi: 10.1016/j.forsciint.2021.111060 34736047

[B34] Kaszubinski S. F. Receveur J. P. Nestle E. D. Pechal J. L. Benbow M. E. (2022). Microbial community succession of submerged bones in an aquatic habitat. J. Forensic Sci. 67, 1565–1578. doi: 10.1111/1556-4029.15036 35349167

[B35] Koricheva J. Gurevitch J. Mengersen K. (2013). Handbook of Meta-Analysis in Ecology and Evolution. Princeton, NJ: Princeton University Press. doi: 10.1515/9781400846184

[B36] Kuroyanagi E. Honda S. Yoshimi S. Sagara N. (1982). The appearance of Hebeloma radicosum from a buried cat carcass. Trans. Mycol. Soc. Japan. 23, 485–488.

[B37] Leite-Jr D. P. Dantas E. S. Nascimento D. C. Correa H. S. Felippe P. A. Pires R. A. . (2019). Action of fauna and flora on the cadaveric phenomena observed in the carcass of *Sus scrofa* (Linnaeus-Suidae) in the wild area Brazilian savannah of the central region-Brazil. Forensic Res. Criminol. Int. J. 7, 185–199. doi: 10.15406/frcij.07.00285

[B38] Mason A. R. (2022). An investigation of intrinsic and extrinsic factors that influence soil microbial succession during human decomposition [PhD dissertation]. University of Tennessee, Knoxville, TN, United States.

[B39] Mason A. R. McKee-Zech H. S. Steadman D. W. DeBruyn J. M. (2024). Environmental predictors impact microbial-based postmortem interval (PMI) estimation models within human decomposition soils. PLoS One 19:e0311906. doi: 10.1371/journal.pone.0311906 39392823 PMC11469530

[B40] Metcalf J. L. Carter D. O. Knight R. (2014). Characterization of Bacterial and Microbial Eukaryotic Communities (Including Fungal) Associated with Corpse Decomposition Using Next Generation Sequencing. Final Report. Award Number: 2011-DN-BX-K533. Washington, DC: U.S. Department of Justice. Document No. 248523.

[B41] Metcalf J. L. Xu Z. Z. Weiss S. Lax S. Van Treuren W. Hyde E. R. . (2016). Microbial community assembly and metabolic function during mammalian corpse decomposition. Science 351, 158–162. doi: 10.1126/science.aad2646 26657285

[B42] Miller R. N. Hilton O. K. (1986). New and interesting agarics from Western Australia. Sydowia 39, 126–135.

[B43] Nilsson R. H. Larsson K. H. Taylor A. F. S. Bengtsson-Palme J. Jeppesen T. S. Schigel D. . (2019). The UNITE database for molecular identification of fungi: handling dark taxa and parallel taxonomic classifications. Nucleic Acids Res. 47, D259–D264. doi: 10.1093/nar/gky1022 30371820 PMC6324048

[B44] Pacioni G. Leonardi M. Aimola P. (2015). “Secondary metabolites produced by fungi affecting human health,” in Biosynthesis and Molecular Genetics of Fungal Secondary Metabolites (New York, NY: Springer), 1–24.

[B45] Pechal J. L. Schmidt C. J. Jordan H. R. Benbow M. E. (2018). A large-scale survey of the postmortem human microbiome, and its potential to provide insight into the living health condition. Sci. Rep. 8:5724. doi: 10.1038/s41598-018-23989-w 29636512 PMC5893548

[B46] Piepenbring M. Baschien C. Hoffmann L. Olesiuk M. Gehrels D. Amendt J. . (2025). Exploring the diversity of culturable fungi on corpses for forensic applications. Mycol. Prog. 24, 1–5. doi: 10.1007/s11557-024-02021-8

[B47] Procopio N. Ghignone S. Voyron S. Chiapello M. Williams A. Chamberlain A. . (2020). Soil fungal communities investigated by metabarcoding within simulated forensic burial contexts. Front. Microbiol. 11:1686. doi: 10.3389/fmicb.2020.01686 32793158 PMC7393272

[B48] Raje S. C. Bhagat D. S. Nimbalkar R. K. Shejul S. K. Bumbrah G. S. Sankhla M. S. . (2022). Contributions and current trends of forensic botany in crime scene investigation. Forensic Sci. J. 21, 1–2. doi: 10.6593/FSJ.202212_21(1).0001

[B49] Sagara N. (1973). Proteophilous fungi and fireplace fungi. Trans. Mycol. Soc. Japan 14, 41–46.

[B50] Sagara N. (1975a). Ammonia fungi: a chemoecological grouping of terrestrial fungi. Contrib. Biol. Lab. Kyoto University. 24, 205–290.

[B51] Sagara N. (1975b). Occurrence of *Laccaria proxima* under carcasses. Trans. Brit. Mycol. Soc. 65, 172–174.

[B52] Sagara N. (1976). Presence of a buried mammalian carcass indicated by fungal fruiting bodies. Nature 262:816. doi: 10.1038/262816a0958460

[B53] Sagara N. (1981). Occurrence of *Laccaria proxima* in the grave site of a cat. Trans. Mycol. Soc. Japan. 22, 271–275. doi: 10.5112/jjlp.22.271

[B54] Sagara N. (1989). European record of the presence of a mole's nest indicated by a particular fungus. Mammalia 53, 301–305. doi: 10.1515/mamm.1989.53.2.301

[B55] Sagara N. (1992). “Experimental disturbances and epigeous fungi,” in The Fungal Community: Its Organization and Role in the Ecosystem, eds. G. C. Carroll, and D. T. Wicklow (Kyoto: CRC Press), 427–454.

[B56] Sagara N. (1995). Association of ectomycorrhizal fungi with decomposed animal wastes in forest habitats: a cleaning symbiosis? Can. J. Bot. 73, 1423–1433. doi: 10.1139/b95-406

[B57] Sagara N. Abe H. Okabe H. (1993a). The persistence of moles in nesting at the same site as indicated by mushroom fruiting and nest reconstruction. Can. J. Bot. 71, 1690–1693. doi: 10.1139/z93-237

[B58] Sagara N. Honda S. Kuroyanagi E. Takayama S. (1981). The occurrence of *Hebeloma radicosum* on the dung-deposited burrows of *Urotrichus talpoides* (shrew mole). *Trans. Mycol. Soc. Japan*. 22, 441–455.

[B59] Sagara N. Hongo T. Murakami Y. Hashimoto T. Nagamasu H. Fukiharu T. . (2000). *Hebeloma radicosoides* sp. nov., an agaric belonging to the chemoecological group ammonia fungi. Mycol. Res. 104, 1017–1024. doi: 10.1017/S0953756299002439

[B60] Sagara N. Kitamoto Y. Nishio R. Yoshimi S. (1985). Association of two *Hebeloma* species with decomposed nests of vespine wasps. Trans. Brit. Mycol. Soc. 84, 349–352. doi: 10.1016/S0007-1536(85)80090-5

[B61] Sagara N. Okabe H. Kikichu J. (1993b). Occurrence of an agaric fungus *Hebeloma* on the underground nest of wood mouse. Trans. Mycol. Soc. Japan 34, 315–322.

[B62] Sagara N. Yamanaka T. Tibbett M. (2008). “Soil fungi associated with graves and latrines: Toward a forensic mycology,” in Soil Analysis in Forensic Taphonomy (Boca Raton, FL: CRC Press), 67–107. doi: 10.1201/9781420069921.ch4

[B63] Schoch C. L. Seifert K. A. Huhndorf S. Robert V. Spouge J. L. Levesque C. A. . (2012). Nuclear ribosomal internal transcribed spacer (ITS) region as a universal DNA barcode marker for Fungi. Proc. Natl. Acad. Sci. U. S. A. 109, 6241–6246. doi: 10.1073/pnas.1117018109 22454494 PMC3341068

[B64] Schwarz P. Dannaoui E. Gehl A. Felske-Zech H. Birngruber C. G. Dettmeyer R. B. . (2015). Molecular identification of fungi found on decomposed human bodies in forensic autopsy cases. Int. J. Leg. Med. 129, 785–791. doi: 10.1007/s00414-014-1118-6 25398636

[B65] Sidrim J. J. Moreira Filho R. E. Cordeiro R. A. Rocha M. F. Caetano E. P. Monteiro A. J. . (2010). Fungal microbiota dynamics as a postmortem investigation tool: focus on *Aspergillus, Penicillium* and *Candida* species. J. Appl. Microbiol. 108, 1751–1756. doi: 10.1111/j.1365-2672.2009.04573.x 19863685

[B66] Šimonovičová A. Kraková L. Pangallo D. Majorošová M. Piecková E. Bodoriková S. . (2015). Fungi on mummified human remains and in the indoor air in the Kuffner family crypt in Sládkovičovo (Slovakia). Int. Biodeterior. Biodegradation. 99, 157–164. doi: 10.1016/j.ibiod.12.011

[B67] Spychała K. Piecuch A. Szleszkowski Ł. Kadej M. Ogórek R. (2024). Microscopic fungi on the corpse - promising tool requiring further research. Forensic Sci. Int. 361:112129. doi: 10.1016/j.forsciint.2024.112129 38986228

[B68] Stamets P. (2005). Mycelium Running: How Mushrooms Can Help Save the World. Berkeley, CA: Ten Speed Press. doi: 10.1016/j.explore.12.011

[B69] Szelecz I. Lösch S. Seppey C. V. Lara E. Singer D. Sorge F. . (2018). Comparative analysis of bones, mites, soil chemistry, nematodes and soil micro-eukaryotes from a suspected homicide to estimate the post-mortem interval. Sci. Rep. 8:25. doi: 10.1038/s41598-017-18179-z 29311698 PMC5758714

[B70] Taylor L. S. (2020). A high resolution study of long-term vertebrate decomposition in human and animal model systems [PhD dissertation]. University of Tennessee, Knoxville, TN, United States.

[B71] Tedersoo L. Bahram M. Põlme S. Kõljalg U. Yorou N. S. Wijesundera R. . (2014). Global diversity and geography of soil fungi. Science 346:1256688. doi: 10.1126/science.1256688 25430773

[B72] Tibbett M. Carter D. O. (2003). Mushrooms and taphonomy: the fungi that mark woodland graves. Mycologist 17, 20–24. doi: 10.1017/S0269-915X(03)00115-0

[B73] Tranchida M. C. Cabello M. N. (2017). The mycology as forensics tool. Adv. Tech. Biol. Med. 5:226. doi: 10.4172/2379-1764.1000226

[B74] Tranchida M. C. Pelizza S. A. Elíades L. A. (2021). The use of fungi in forensic science, a brief overview. Can. Soc. Forensic Sci. J. 54, 35–48. doi: 10.1080/00085030.2020.1869390

[B75] Vidona W. B. Adetunji C. O. Willy-Vidona C. (2025). “The influence of mushroom on the taphonomic process of cadaver,” in Mushroom Biotechnology for Improved Agriculture and Human Health (Hoboken, NJ: Wiley), 307–315. doi: 10.1002/9781394212699.ch14

[B76] Xue C. Shen J. Song J. Yang J. Zhang S. Wang J. (2025). Pulmonary infection caused by Schizophyllum commune: a case study. Front. Med. 12:1475896. doi: 10.3389/fmed.2025.1475896 PMC12206706 40589965

